# Implementation of a comprehensive program including psycho-social and treatment literacy activities to improve adherence to HIV care and treatment for a pediatric population in Kenya

**DOI:** 10.1186/1471-2431-8-52

**Published:** 2008-11-21

**Authors:** Joelle Van Winghem, Barbara Telfer, Tony Reid, Judith Ouko, Angela Mutunga, Zaina Jama, Shobha Vakil

**Affiliations:** 1 Operational Cell Belgium, Médecins Sans Frontières, Nairobi, Kenya; 2 Operational Research Centre, Médecins Sans Frontières, Brussels, Belgium; 3 Technical advisor, ART Program, Government of Kenya Ministry of Health, National AIDS and STD Control Programme (NASCOP), Nairobi, Kenya

## Abstract

**Background:**

To achieve good clinical outcomes with HAART, patient adherence to treatment and care is a key factor. Since the literature on how to care for pediatric HIV patients is limited, we describe here adherence interventions implemented in our comprehensive care program in a resource-limited setting in Kenya.

**Methods:**

We based our program on factors reported to influence adherence to HIV care and treatment. We describe, in detail, our program with respect to how we adapted our clinical settings, implemented psycho-social support activities for children and their caregivers and developed treatment literacy for children and teenagers living with HIV/AIDS.

**Results:**

This paper focused on the details of the program, with the treatment outcomes as secondary. However, our program appeared to have been effective; for 648 children under 15 years of age who were started on HAART, the Kaplan-Meier mortality survival estimate was 95.27% (95%CI 93.16–96.74) at 12 months after the time of initiation of HAART.

**Conclusion:**

Our model of pediatric HIV/AIDS care, focused on a child-centered approach with inclusion of caregivers and extended family, addressed the main factors influencing treatment adherence. It appeared to produce good results and is replicable in resource-limited settings.

## Background

While the overall prevalence of Human Immunodeficiency Virus (HIV) infection in Kenya was reported to have declined to 5.1% in 2006, an estimated 102,000 children under 15 years of age were living with HIV, of whom about 6000 received highly active antiretroviral therapy (HAART).[1,2] In resource limited settings, HAART has produced good results in the treatment of HIV in children.[3-7] In Kenya, increased access to free HIV-rapid testing, PCR testing and, since June 2006, free HAART, has reduced mortality among children living with HIV.[2]

To achieve good clinical outcomes from HAART, patient adherence to treatment and care is a key feature. [8] Adherence is also important to reduce the risk of development of drug resistant strains of HIV.[8] For children living with HIV, a number of studies have emphasized the importance of patient and caregiver psychosocial support to optimise adherence to treatment and care. [9-13] Children are vulnerable and feel stress differently from adults. For instance, they are often dependent on caregivers, orphaned or have lost at least one parent.[2,14] Many live in foster families, and there are difficulties with disclosure of their HIV-positive status. Children living with HIV often report experiencing discrimination in school, church, the community or family, and they have fears of the future. [14,15]

### Predictors of adherence to treatment and care

Factors that predict problems with adherence to treatment include patient related factors, medication factors, the relationship between the patient and health care provider, and the system of care. [8] We discuss briefly the first three. Patient related factors include patient resources, knowledge, attitudes, beliefs, perceptions, expectations and psychosocial issues, all of which influence behaviour, the critical link between treatment and outcome. [8] Adherence can also be influenced by socioeconomic circumstances that may lead to a situation of having to choose between competing priorities, paying the transport fee to go to the clinic or buying food for the family. [8] Medication related factors include the complexity of the medical regimen, the dosage and duration of treatment, side effects, and availability of medical support and patient education. [8] The quality of the relationship between patient and health care provider in the management of a chronic disease such as HIV is important to ensure effective communication, continuity of care, confidence and trust so that they are seen as partners in care and not as authoritarian figures. [8,13]

Adherence is a dynamic and complex process that is influenced by the interplay of these and other factors. [8] For children living with HIV, strategies to optimise adherence to HIV treatment and care require regular review and adaptation based on the individual needs and growth of a child. [16,17] Interventions to optimise treatment adherence among children living with HIV, such as psychosocial support activities, have seldom been described in detail in the literature, so they can be reproduced. We describe here in detail adherence interventions in our pediatric HIV comprehensive care program in a resource-limited setting.

### The setting of our pediatric HIV/AIDS care program

Since 1997 MSF-Belgium has been providing care and treatment for people living with HIV/AIDS in Nairobi, Kenya, in collaboration with the Ministry of Health (MOH). The program has provided free HIV care and treatment to outpatients at the only public district hospital in Nairobi Province and to patients at three clinics in the informal settlement of Kibera. The comprehensive care package included counselling and testing, management of opportunistic infections, HAART assessment and treatment (since 2003), psychosocial support, nutritional support and treatment literacy training. In two of the Kibera clinics HIV and tuberculosis (TB) care were integrated into comprehensive primary health care services. MSFB has also conducted a range of training and health promotion activities for patients, local communities and health care workers, to increase the capacity of these groups to better understand and manage HIV/AIDS.

**Figure 1:**
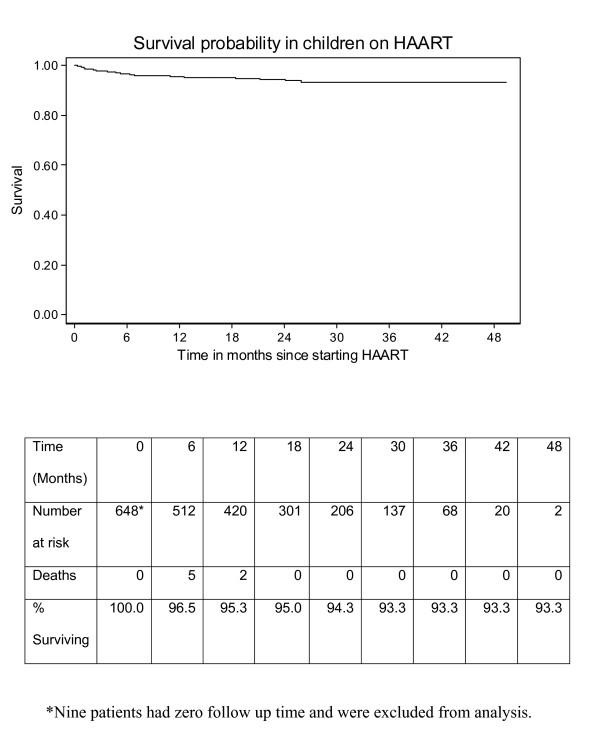
Kaplan-Meier survival curve of children placed on Highly Active Anti-Retroviral Treatment in Nairobi, Kenya.

To summarise the profile of our pediatric cohort, as of 30 November 2007, 1205 HIV-infected and HAART-naive children under 15 years of age had been enrolled in our HIV program, 657 (55%) of whom were initiated on HAART (Table 1). Of the 657 children initiated on HAART, 452 (69%) were still on HAART and in active care on 30 November 2007 (attended at least one appointment since 1 August 2007). Kaplan-Meier survival methods were used to estimate the probability of survival from the time of initiation of HAART to death. Date of death was taken as the endpoint for those patients who died. A patient was considered lost to follow up if they were not confirmed dead, had not been transferred out, and had not attended the clinic in the four months prior to the date of analysis (no clinic attendance on or since 1 August 2007). Patients lost to follow up were censored at the time of last appointment to a clinic. Patients transferred out to another service provider were censored at the time of transfer. All other patients were censored at the time of last clinic visit up to 30 November 2007. Analysis was conducted in STATA version 8.2 (StataCorp, 4905 Lakeway Drive College Station, Texas, 77845, USA). The Kaplan-Meier mortality survival estimate at 12 months was 95.3% (95%CI 93.2–96.7) and at 24 months was 94.3% (95%CI 91.9–96.1) (Figure 1). This is comparable to mortality survival estimates reported among a pediatric HAART cohort from South Africa. [4]

**Table 1:** Characteristics of Antiretroviral-naïve children (1 January 2003 – 30 November 2007)

**Characteristics**	**Number (%)**
Number ever enrolled in HIV care	1205
Sex	
- *Female*	638 (53.0%)
Age at time of enrolment	
- *< 3 years*	398 (33.0%)
- *3 – 5 years*	371 (30.8%)
- *6 – 8 years*	231 (19.2%)
- *9 – 14 years*	205 (17.0%)
- *Median age in years (IQR*^*a*^)	4.88 (2.0–7.8)
Length of time in program (years)	
- *Average*	1.28
- *Median*	0.95
- *IQR*	0.2–2.1
Number initiated on HAART^b^	657 (54.5%)
Age when started HAART	
- *< 3 years*	147 (22.4%)
- *3 – 5 years*	204 (31.1%)
- *6 – 8 years*	153 (23.3%)
- *9 – 14 years*	153 (23.3%)
- *Median age in years (IQR)*	5.50 (3.2–8.7)
Sex of patients placed on HAART	
- *Female*	327 (49.8%)
Length of time on HAART (years)	
- *Average*	1.47
- *Median*	1.36
- *IQR*	0.6–2.2
Outcomes of patients on HAART on 30 November 2007:	657
- *On treatment and followed*	452 (68.8%)
- *Dead*	32 (4.9%)
- *Transferred out*	106 (16.1%)
- *Lost to follow up (did not attend clinics since 1 August 07)*	67 (10.2%)
Number switched to second line therapy	7 (1.1%)

^a. ^Interquartile range

^b. ^Highly Active Anti-Retroviral Treatment

Through our experience of operating a pediatric HAART program in a resource-limited setting with a high HIV prevalence, we noticed that the psychological impact of HIV on children was often overlooked. We initially observed a high loss to follow up in our pediatric HAART cohort. In response to these challenges and to scale up and strengthen our program we adopted a more a holistic approach to the management of child and teenage patients. An important feature has been the constant adaptation over time of our program, based on the needs of patients and experience in the field.

### Adaptation of the system of care

In 2005 a multidisciplinary working group comprising clinicians, counsellors, social workers, and health promotion officers identified, along the medical care pathway from testing to treatment and follow up, critical points where predictors of adherence for children and teenagers could be potentially addressed. Our aim was to strengthen the adherence of children to treatment and care, as well as to scale up testing and enrolment of HIV-infected children into the program. The medical care pathway and treatments for children living with HIV in our program were systematised and in accordance with the World Health Organization (WHO) and Kenyan National Guidelines, and updated as required.[18,19]

### Critical points along the medical care pathway where predictors of adherence could be addressed

#### 1. HIV testing and disclosure of the results to a child

In our project the disclosure of the HIV positive status of a child by a caregiver was a major challenge, and became a focal point for psychosocial support to both parties. The absence of disclosure has been reported as a risk factor for non-adherence, and we observed this is in our program. [9,13]

We emphasized the family approach, by asking for each adult patient, at every visit, about their child's health and HIV status. For each child testing HIV positive, we proposed that siblings and parents be tested. Families were given appointments for the same day as much as possible.

The actual timing of disclosure was not recorded, but according to the counsellors in charge of the children's and teenagers' groups, the average age where disclosure was carried out was between 8–12 years of age with a "best" age at 9 years. This appeared to be an appropriate age since the child had a good understanding of the disease and that he or she had to receive treatment for life. The timing of disclosure also depended on other factors such as the educational background of the child, his or her social exposure and level of understanding.

#### 2. Patient enrolment in the clinic, HAART therapy assessment, preparation, adherence and follow up

The following stages of patient management were considered important to target with psychosocial activities and other strategies to support adherence to treatment and care.

Once a child was confirmed to be HIV-positive, a unique patient file was opened, and the first medical examination was conducted, including a CD4 count. If the child qualified for HAART, a process of multidisciplinary assessment was commenced: the counsellor, social worker, nutritionist and medical staff assessed the patient. The multidisciplinary team (MDT) together with the patient and/or caregiver developed an individual plan for preparation and initiation of treatment.

If HAART was commenced, the child came back after two weeks to be assessed for HAART tolerance, adherence counselling and other psychosocial and health concerns. Patient progress was regularly monitored with an individually-tailored appointment schedule. If there were any concerns regarding a patient, the MDT reviewed the case and then discussed the issues with the caregiver and the child, to include them in the decision-making process.

We set the following goals for psychosocial support and other activities for child and teenage patients: to help patients to cope better with their HIV status and disease, understand and accept the necessity of life-long and sometimes intense treatment, improve quality of life, avoid stress and suffering, and to support the child's growth and development. To ensure their active participation in decisions about their own health care, the child and teenage patient were placed at the centre of the psychosocial activities. The goals of psychosocial support and other activities for caregivers (and where possible the entire family) were to assist them to understand and accept HIV and the need for prolonged treatment and follow-up and to manage the child in the same manner as HIV-negative siblings.

Adherence was assessed at every visit by self report, pill count and pharmacy records. It is interesting to note from that the patients' side that they sometimes felt as though we didn't trust them when we kept asking about adherence and counting pills. Measurement of CD4 counts were conducted at regular intervals for each patient, and viral load (VL) studies were performed on a case by case basis as required (unpublished data).

### Interventions to promote adherence

Based on factors influencing adherence, we implemented a range of interventions (Table 2). The interventions can be described along three axes: adaptation of our system of care, psychosocial support activities and capacity building activities.

**Table 2:** Interventions to promote adherence to a pediatric HAART program, Nairobi

**Factors predicting adherence to treatment and care**	**System adaptation**	**Psycho-social support**	**Capacity building**
**Patient-related factors**	**Child friendly clinics with**: - Playground	- Social support network to refer patient to according to the needs and demands.	- Treatment literacy training
	- A dedicated pediatric clinic day	- Development of material (fairytale, booklet)	- Hero book training
	- Pediatric waiting bay with toys, video, games.	- Support groups	- Attitude training
	- Pediatric counselling room	- Individuals therapy session	- Community based campaigns to promote awareness and understanding of the specific plight of children living with HIV/AIDS
	- Testing and counselling decentralised in the ward with special session to help the coping with disclosure of positive status	- Financial support for bus fare	
	- 3 target groups: children, teenagers and caregivers	- Referral to Post Test Clubs	
		- Art therapy	
		- Sand therapy	
		- Individual counselling tailored schedule	
**Therapy related factors**	- Pill boxes		- Treatment literacy training
	- Tick sheets		
	- Enrolment of children born from a positive mother until HIV status is confirmed		
	- Malnutrition screening		
	- Provision of supplementary and therapeutic food		
	- Referral to a Pediatrician		
**Patient-provider relationship factors**	- Childrens' Fun Day		**Staff training in**:
			- HIV medicine
	- Training of young peer PLWHA in basic counselling and in training of the trainer on a range of curricula		- Pediatric HAART
			- Basic counselling
			- Attitude training
			- Treatment literacy
			- Counsellors trained in various techniques for counselling (Hero book, play therapy, and training of the trainer for treatment literacy)
			- PLWHA peer counsellor trained in basic counselling skills, treatment literacy and ToT for TL

#### Axis 1

First, **we tailored our system of care **to better meet the needs of child and teenage patients through:

##### a) Creation of child-friendly clinic environments

In each clinic a special room was dedicated for children and furnished with games, toys, videos and colourful painted walls. At the hospital based clinic a playground was constructed adjacent to the clinic. One day per week was dedicated to being a pediatric clinic day. On this day children in each clinic were gathered in the pediatric room for group activities, led by young adult peer educators with the help of a counsellor and sometimes a volunteer (for example, a teacher living with HIV). During the pediatric clinic day a pediatrician was available to see complicated medical cases.

##### b) Strengthening the family approach

Family-centered care was the key theme in this program. Adult and children were enrolled in the same clinic. When one family member tested positive, the health staff (counsellors and clinicians) took every opportunity to get other members tested. Adult patients were usually only enrolled if they were in WHO Clinical Stages 3 or 4 however, if they were in Stage 1 or 2, and had a child in the clinic, they were automatically enrolled. Whenever a child had an appointment, the parent was seen as well; the bookings were synchronized whenever possible.

On pediatric days, parents and caregivers were gathered in separate groups for discussion. Parents with unknown or negative status were grouped together and patient-parents formed their own group. Issues were discussed such as importance of knowing your status, disclosure of status, and how to care for a child living with HIV, especially when you had other children. For parents or caregivers of unknown or HIV negative status, treatment literacy covered some basics about home-based care for positive children as well as the usual subjects related to HAART adherence.

Following the Hero Book training, during which the child creates a kind of memory book that has him function both as the hero, the illustrator and the story teller, thus allowing him to confront particular challenges in his life, time was devoted for an individual meeting including the child, a counsellor and the caregiver to go through the book and answer any questions. This permitted the caregiver and counsellor to gauge how well the child understood their illness.

To address social support issues, we tried to work within a network of other non-governmental organizations to whom we could refer families for shelter, school fees, food support, and other social needs.

The caregivers of orphans had their own issues like feeling unsupported and/or discouraged about caring for a child who was not their own. They had fears about their own children becoming infected from the orphan or the effect of positive discrimination towards the HIV positive child (e.g., giving them special food, a special plate, special attention, etc). All these issues were tackled in open meetings or directly in the support groups and treatment literacy sessions whose curriculum had been tailored to address all these issues.

The orphans in custody of the grandparents presented other challenges. Often the grandparents were illiterate and didn't have a stable financial income (as they were working anymore). For these caregivers, we met with them individually, to educate them on the disease and implications. We provided transport fees and referred them to other partners for social support.

##### c) Strengthening the system for tracing patients who miss appointments

Every patient who missed an appointment was traced by a phone call, home or hospital visit.

##### d) 'Child/teenager Fun Days'

During every school holiday period on Saturdays, groups of about 40 children and 20 staff (including reception staff, data entry clerks, doctors, social workers, peer educators) spent a social day out together. Teenage patients and a limited number of caregivers also participated. The fun days were comprised of visits to child-friendly sites (such as a giraffe centre, animal orphanage, and fun parks), activities (such as Olympic games, live music and dancing, Christmas activities) in schools that agreed to lend us their fields, and a shared meal. Fun days have strengthened the relationships between patients and staff and provided them with an opportunity to talk and socialise outside of the clinic and have fun together. Fun days have also given children and caregivers the chance to meet peers and form social networks. Another important aim was to take the children out of their daily lives just to have fun.

##### e) Setting three target groups for psychosocial support and capacity-building interventions: children, teenagers and caregivers

We set these target groups to ensure we tailored adherence interventions to the unique needs of each group. Children were still quite dependent and needed parenting. Teenage patients had their own particular issues regarding the beginning of their own sexual lives, fear of transmitting the virus, fear of not being able to get married and have children, feelings of depression or even suicide. Caregivers had their own issues either due to being HIV positive themselves or trying to integrate a HIV positive child into their family.

##### f) Pillboxes and tick sheets to assist patients with taking treatment

To help the child taking their pills on time, a system of tick sheets and pillboxes was initiated. For some children, a watch with an alarm was provided.

#### Axis 2

Second, **we developed and implemented a range of psychosocial support activities and tools **for children, teenagers and their caregivers, including:

##### a) A fairytale titled "*Thanks ARVS*"

The fairytale tells how germs (such as the virus causing flu) and the HIV virus were spread in an animal village by a wicked hyena, causing the villagers to become sick and die. It explained how normally the magic force (immunity cells) in their bodies could fight the germs and how HIV weakened that magic force by using them to replicate themselves in the body. Fortunately, Uncle Lion and Aunt Elephant found a treatment (HAART) to stop the virus replicating and destroying the magic force. They give advice on how to stay healthy and on the importance of supporting each other to adhere to treatment and to cope with challenges, such as the bad taste of some pills that can be hard to swallow. Developed by a counsellor in the MSF-Belgium Thailand HIV project, this fairytale was adapted to the Kenyan context and published in English and Kiswahili languages [Additional file 1]. The fairytale was used as a communication tool and was aimed at creating a positive understanding of HIV, emphasizing basic concepts about the disease, treatment and how to maintain health. At the end of the book were questions about the fairytale, but also about the reader's own story. The fairytale helped children verbalise questions and concerns about the story, but also to progressively relate themselves to that story and recognise their own situation.

This tool has been used by counsellors at the stage of pre-HAART assessment; the counsellor read through the story with the child, the child then took the book home, and at the second HAART-assessment counselling session, the counsellor went through the questions to assess if the child has understood what it was about (long term disease and treatment, importance of taking the pills everyday, importance of healthy and positive living). The fairytale has also proven useful in assisting caregivers and counsellors to disclose to a child their HIV-positive status, through reading the story and answering the child's questions, for example, "*Oh the giraffe is taking pills morning and evening like me; do we have the same disease?*...".

##### b) A booklet for teenagers titled "*All you need to know about HIV and ARVS *sYouth Booklet"

This is a pocket size booklet containing basic information about HIV, AIDS, opportunistic infections (OIs), HAART, nutrition and positive living attitudes, illustrated in a colourful layout with drawings by a young Kenyan artist [Additional file 2]. The drawings illustrate the fight between Masaai warriors (CD4) and the virus. It was often used with teenage patients in the pre-HAART assessment counselling sessions; the counsellor went through the booklet with the patient, the patient then took the booklet home, and during the second HAART assessment counselling session, the counsellor answered and asked questions to assess the level of understanding of the youth. The booklet was also a useful tool for participants of treatment literacy training.

##### c) Individual counselling and specialised counselling techniques

Individual counselling was strengthened through the adoption of special psychotherapy techniques. No specific behavioural model was used, but the counsellors were trained by the Kenyan Association of Professional Counsellors (KAPC) in association with Durham University (UK) on child counselling techniques.

Children seldom spontaneously mention their feelings, so techniques of drawing or playing were used to facilitate discussion. Counsellors were trained in these techniques. ***Sand therapy ***was used as a therapeutic technique employing a sand tray, a source of water, and miniatures of people, animals, buildings, bridges, vehicles, furniture, food, plants and rocks, with which the child could build scenes on the sand. Sand therapy was used to gain access to the content of the unconscious.[20,21] The counsellor observed the play and how a child expressed themselves through manipulation of the miniature objects and the creation of a diorama, without intrusion. A review of the scene took place later with the child, and the counsellor made notes that were retained in the patient file so that a session to session assessment could be made of the psychotherapeutic process.[20,21] Counsellor's also implemented ***art-therapy***, with drawing and clay modelling as the medium in a similar, observational manner, during individual counselling sessions.

These different psychotherapy techniques could be used at any stage after a patient was enrolled in the clinic, whether or not the child was on HAART. These techniques were used to deal with patient concerns such as coping with their HIV-positive status, stigma, discrimination, death of a family member or fear of their own death. Children were identified by the counsellors for these specific psychological interventions through individual counselling sessions, group activities (hero book, treatment literacy, support groups) or disclosure sessions. The selection of these children was left to the counsellor's discretion. For example, during a Hero book session with one particular child, while addressing the issues of family tree, the child burst into tears while remembering his deceased father and then was very quiet and sad for the rest of the session. This was new information since this child had been followed in normal sessions without this issue being raised. This child was assessed by the counsellor to be a good candidate for individual counselling.

##### d) Support groups for children and teenage patients

Support groups for children (usually 10 to 13 years of age) and teenagers (usually 14 to 18 years of age) were commenced and each comprised of up to 15 participants. Each support group was lead by two counsellors. The decision to join a support group was a joint one between the patient and a counsellor.

The children's support groups were proposed by the children themselves, following one of the treatment literacy trainings. They felt it was important to be part of a support group, and planned to meet monthly. Within the group sessions children shared their experiences, especially about stigma and discrimination in their family and at school. The counsellors facilitated the discussion to assist the children to talk freely without feeling ashamed or guilty about their experiences. During support group sessions, children told each other their stories, offered each other support, and proposed solutions to cope with issues raised, based on their own experiences. Our experience has been that children greatly appreciate belonging to a group and the strength and support they derive from the group. On two occasions the children consented to the anonymous recording and transcription of children's stories discussed in the support groups, and this material has been used in forums to raise awareness about stigma and discrimination experienced by children living with HIV. The children's support group began with 15 participants in 2007 and increased to 59 by mid-2008.

The teenager's group support was proposed by a young counsellor who was concerned about the feelings of loneliness and desperation he had observed among teenage patients. Teenage patients were enthusiastic about the idea of a support group, and relieved to know that they were not alone in having difficulty coping with their diagnosis. The teenage support group called themselves "Les Anges de la vie" (angels of life) and utilised t-shirts bearing this slogan to prompt people to ask what the slogan meant. This gave the teenagers a chance to share their story with others. Within the group the participants shared stories and went on excursions to have fun with their peers. The support group has helped to develop the teenagers to a stage where many of them wish to be open about their status and their experiences of stigma and discrimination. The teenager support group began with 12 participants in 2007 and increased to 36 regular participants by mid-2008.

The following anecdote was reported by counsellors and gives an indication of the potential benefits of support groups: A teenage girl was on first line treatment but not improving, (VL > 100,000) despite her claim that she was taking the pills. A switch to second line treatment was considered. However, when she started to talk about feeling lonely and even contemplating suicide, her counsellor encouraged her to join the support group. While attending the support group, she related in her own words how she was in the process of accepting herself. This led to a real improvement in her adherence and she remained on first line regimen, since her VL had dropped to less than 600. She continued to attend every meeting of the support group, and even brought two friends from school who were HIV positive and received their drugs elsewhere.

##### e) Mini groups for caregivers

We established support (mini) groups for caregivers which were held on each dedicated pediatric clinic day. Mini-groups were 30 to 45 minutes in duration and took place while caregivers waited for their child to be called for a consultation. Mini groups comprised of about 12 to 15 caregivers (regardless of HIV status) and were led by a peer counsellor living with HIV who had been trained in basic counselling skills. Depending on the group dynamic, the peer counsellor started the session by sharing their personal experience as a young person living with HIV, then opened up the discussion to the group for questions and sharing of experiences among the group. If the group had met in the past, the peer counsellor chose a theme and the group discussed experiences on that theme. Time was given at the end of each session to discuss any other issues. Important themes included disclosure, stigma and discrimination, nutrition and the importance of honouring appointments.

##### f) Social support

Financial assistance was provided on a case-by-case basis (for example, to meet transport costs for appointments or cover costs of tests to be conducted outside the clinic). Members of the MDT who identified a patient in extreme need referred the patient to one of the social workers for assessment. The social workers established a social support network system whereby patients with specific needs were referred to other organisations for support as school fees, school materials and uniforms, food, and legal issues.

#### Axis 3

Third, **we developed and implemented a range of capacity building interventions **tailored to the needs of child and teenage patients and caregivers. These interventions included:

##### a) Treatment Literacy Training

UNESCO/WHO have identified treatment education as "...forming the bridge between the provision of treatment and the preparation and involvement of people and communities in comprehensive responses to HIV and AIDS". [[22]. p.5] Based on this philosophy, we developed a three-day theoretical training in treatment literacy that was regularly updated by the MDT. The curriculum has been tailored to meet the unique needs and capacities of each of the target groups.

Treatment literacy training was three days in duration for caregivers, and four, three-quarter days (8 am to 2 pm) for child and teenage patients as their concentration was reduced considerably after lunch. Each course consisted of 10 to 15 participants led by two counsellors. Peer counsellors living with HIV and medical staff joined in for training on specific topics. An interactive approach to training was used. Approximately 130 children and 35 caregivers were trained in 3 years.

The aim of treatment literacy training for child and teenage patients was to provide knowledge and skills to understand and manage (to the best of their capacities) their disease, treatment and broader health issues, and to equip them with tools to take some responsibility for their own health. For caregivers, the aim was to provide knowledge and skills to better understand the disease process in their children and its consequences. Issues addressed included knowledge, attitudes and beliefs towards HIV status disclosure, HIV and associated treatment and care, opportunistic infections, nutrition, home based care, and the development of communication skills. For caregivers living with HIV, treatment literacy training for adults was also provided.

##### b) Hero book training

The 'Hero book' was a form of memory book in which the child/teenager was the hero and the author. It included a series of drawing exercises and autobiographic story telling. It assisted children to think about and express their problems and challenges and to develop ways of solving them. The Hero book was developed under the umbrella of the 10 Million Memory Projects, a psychosocial support initiative by the Regional Psycho Social Support Initiative (REPSSI) and partners [21]. Details of Hero book training can be found at . [23]

Hero book training comprised a week of group sessions with a maximum of ten participants of a similar age. Our first Hero book trainings were led by external artists and counsellors. More recently, three to four of our trained counsellors lead each Hero book training. Children did not have to know their HIV status to attend. Costs of transport, food and materials were covered.

##### c) Clinic-based staff training and support

Staff of all disciplines participated in training on a range of topics including HIV medicine, pediatric HAART, attitude training, treatment literacy, basic counselling and communication skills and advanced counselling techniques. Staff were supervised and attended regular profession-based and MDT meetings. Staff who required counselling for work related issues were referred to a confidential, external counsellor who attended the clinics once per week.

##### d). Increased involvement of patients and people living with HIV/AIDS in clinic and community based activities

Four patients were trained as peer educators and counsellors and integrated into the program's work force. In a period of 3 years around 280 patients also completed Training of the Trainer (ToT) courses in treatment literacy and 120 in attitude training, and they have been recruited to train others in these areas.

While many of these interventions could be introduced in a flexible manner anywhere along the medical care pathway, some were particularly useful at certain stages. For instance, to target the critical point of HIV testing and disclosure, we utilised the Fairytale, Treatment Literacy Training for caregivers, and individual counselling sessions and support (or mini) group sessions for caregivers. To target the critical points along the path of patient enrolment in the clinic, HAART assessment, preparation, adherence and follow up, the various support activities included treatment literacy for children and teenagers, Hero book training, support groups for the patient, HAART preparation and adherence counselling and specific individual and group counselling activities for children.

## Discussion

This report describes in detail our comprehensive program for providing HIV care and HAART to children in a challenging, resource-limited setting. Attention to the principles of good adherence led us to individualize care for both child/teenage patients and their caregivers, actively address psychosocial needs and build capacity of children and teenagers to engage in their own treatment. We believe that this program helped to realize good treatment outcomes and that our experience would benefit other programs in similar circumstances.

Our description illustrates how it was possible to implement a child-centred approach with full consideration of the child/teenager as the patient-individual. In addition, attention to the child's family was a key component in the program. This approach is important because HIV is a long term condition and HAART is a life long therapy which needs to be strictly followed. From a public health point of view maintaining adherence is the only way of preventing drug resistance. It is also important because children face different types of stress compared to adults. Our program tried to deal with this issue with its flexible approach and adapted tools. Revisiting the principles of adherence, we believe that this program addressed most of them in a realistic manner. Patient-related factors of knowledge, attitudes, beliefs and fears were approached in several ways, from education, to play groups to individual counselling. Basic socioeconomic conditions were cared for by social workers. Difficulties in taking medications were managed by practical tools for pill monitoring and education around their importance. Finally, the relationships between caregivers and patients/family were strengthened in many ways from use of peer counsellors to fun days. We believe our good medical outcomes are due to attention to these key issues.

We have not formally evaluated individual components of the program. It would be difficult to assess which interventions were the most useful for the children or caregivers, as most children participate in several activities concurrently, and the 'package' of interventions is tailored for each individual. We acknowledge that a formal qualitative evaluation of individual interventions would be very useful. There is however, continual communication between patients, caregivers and staff that is fed back to program coordinators, which in turn informs adaptation of the interventions and program. For instance Counsellors reported that in informal group discussions with children, it appeared as though no specific aspects of the program stood out as the best activity, as they considered each aspect to be part of a whole. The children appreciated their own clinic space and the many activities that helped them work out their feelings or just have fun and forget their illness. Similarly, discussions among caregivers in the group sessions revealed problems as the program developed and these were addressed. For example, transport costs were a barrier to attending, and these were paid for once this problem was raised. This informal iterative loop, along with regular patient and program monitoring activities, have been used to informally evaluate aspects of the program.

### Challenges

There were a number of challenges in implementing our program that need to be discussed. The dependence of the child on their caregivers meant that the child's adherence was directly related to the willingness and ability of the caregiver to attend. If the caregiver was not convinced about the need for regular attendance or was unable to accompany the child due to work or illness, adherence suffered. If there were several caregivers, it could be difficult to communicate correctly on the treatment plan. Hence, we made strong efforts to engage the caregivers as key members of the treatment team.

The socio-economic situation of the family was also a challenge, given that transport fees were double every time the child had an appointment (child and caregiver). We addressed this by providing financial assistance for transport on case-by-case basis after social assessment.

Another problem was that pediatric HIV care was still not a priority in the HIV care program; the focus was mainly on adults especially for the adherence counselling support package. It was a challenge to introduce a child-centred approach with inclusion of the caregiver and whenever possible the entire family for care and offer psychosocial support. Eventually, our efforts were recognized, but this problem may be present in other contexts and needs to be anticipated.

Practical concerns included that the size of pillboxes was inconvenient for some regimens (DDI or syrup), and the price of pillboxes was too high. This required additional funding by MSF, and may be an issue of sustainability in government funded programs.

Children were easily bored if the same material was used over and over so that a constant review of the program's operating plans and methods was required to avoid disinterest. As well, fun activities had to take place during school leave or on weekends, creating some scheduling difficulties. For multiple days training, caregivers had to ask permission for leave from work, which was not always granted.

At the structure level, there was limited physical capacity of the clinics to cater for child-specific activities and rooms. Rapid turn over of trained medical staff was a major challenge. It was why we trained more young people living with HIV in counselling and ToT, to shift the tasks from medical staff to volunteers who would be likely to remain long term with the program.

### Ways Forward

To continue to develop the program, we plan to adapt and diversify the range of materials, games, and posters. As well, we hope to make an agreement with a local radio station to have regular show with and for children/teenagers.

In order to ensure continuity and harmonisation of the program activities, we propose having teams of two persons (health promotion and counsellor) "specialised" in pediatric counselling activities, to be responsible for the development or adaptation of strategies and material.

Several other practical suggestions include: having a free telephone line for patient or caregivers to ask their questions or share their concerns; train more young people living with HIV to be counsellors to lead support groups for children and teenagers in their community; target teachers for treatment literacy to reduce stigma and discrimination in the school environment; and develop alternatives to pill boxes, (for example, pill bags made of plastic covered with a local printed fabric).

To scale up the treatment literacy for children/teenagers and caregivers, opening a training center was proposed. The training curriculum would be divided into modules that would take place once a week so the participants could complete the curriculum at their own rhythm according to their availability.

A formal evaluation of the psychosocial support activities for children, teenagers and caregivers is warranted, to try and determine which interventions are most effective in supporting patients to adhere to care and treatment. A patient and program monitoring system is in place and a data management team ensures that a wide range of data is available at routine intervals to assist with program evaluation. At every clinic attendance for each patient, demographic, social, clinical, biological, immunological and laboratory data are recorded and entered into a database, and data are cleaned, analysed and reported on monthly, quarterly, annually and as required. Data are also collected on social work, counselling activities, patient tracing episodes activities, pill count, self report adherence, and other activities. Analysis of these data will be done in further operational research activity and will be submit for publication.

Finally, the issue of sustainability is always present in projects like this. The MoH needs to be convinced that HIV care and treatment for children is a priority. Most of the interventions described here are not expensive, based mostly on careful organization of care and use of volunteers to extend the reach and capacity of the program. We believe that demonstrating good results with low cost to a MoH should be convincing.

## Conclusion

Adherence is the key factor of success of pediatric HAART programs and our experience of focusing on a child-centered approach with inclusion of caregivers and extended family appears to foster this. We believe that our model is feasible in resource-limited settings and hope the details described here will assist other programs in similar contexts.

## Competing interests

The authors declare that they have no competing interests.

## Authors' contributions

JVW (medical doctor) contributed to the conception, design, writing and revision of this paper, and managed the implementation of the holistic approach to paediatric HIV care in the project. BT (epidemiologist) contributed to the conception, design, writing and revision of this paper, and the analysis of data. TR (medical doctor) contributed to the conception, design, writing and revision of this paper. JU, AM and ZJ (counsellors) contributed to the revision of this paper and lead the implementation of the holistic approach to paediatric HIV care in the project. SV (medical doctor) contributed to the conception and support of this paper.

## Pre-publication history

The pre-publication history for this paper can be accessed here:



## Supplementary Material

Additional file 1 **"Thanks ARV"**. A fairytale used as a communication tool and was aimed at creating a positive understanding of HIV, emphasizing basic concepts about the disease, treatment and how to maintain health. Click here for file

Additional file 2 **"*All you need to know about HIV and ARVS *Youth Booklet"**. a pocket size booklet containing basic information about HIV, AIDS, opportunistic infections (OIs), HAART, nutrition and positive living attitudes for teenagers. Click here for file
