# Supplementary material for: Implementation of a comprehensive program including psycho-social and treatment literacy activities to improve adherence to HIV care and treatment for a pediatric population in Kenya
Source: BMC Pediatr. 2008 Nov 21;8:52. doi: 10.1186/1471-2431-8-52 (PMC2613143; doi:10.1186/1471-2431-8-52)
Supplement: Additional file 1 — "Thanks ARV". A fairytale used as a communication tool and was aimed at creating a positive understanding of HIV, emphasizing basic concepts about the disease, treatment and how to maintain health. [file 1471-2431-8-52-S1.pdf]

Information Booklet for Youth

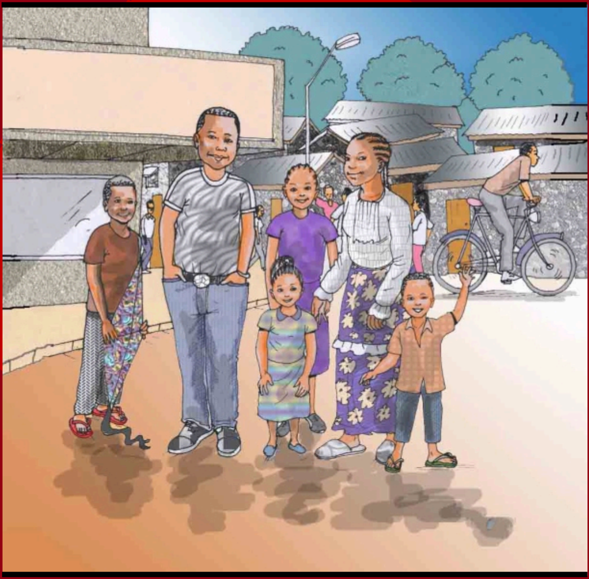

All you need to know about HIV/AIDS & ARV (Anti-Retroviral Drugs)

MEDECINS SANS FRONTIERES

HIV changes your life completely. But stay calm. Nowadays there is a lot that can be done to help you live a healthy, long, positive life and grow up to be a normal adult. Many people are scared. They feel uncertain about what will happen to them and their loved ones. People are also afraid of stigma and discrimination. It means that people who don't know their status or HIV negative people would rather stay away from HIV positive people or try to isolate them and don't realize how very painful this attitude is. But try not to think too much about how others will respond. Think about your own health calmly instead, go to school, continue to see your friend, make new friends, play games and continue to live positively.

ARVs can help you!!

Over a million adults, youth and children around the world take ARVs every day,

YOU too can take them successfully!

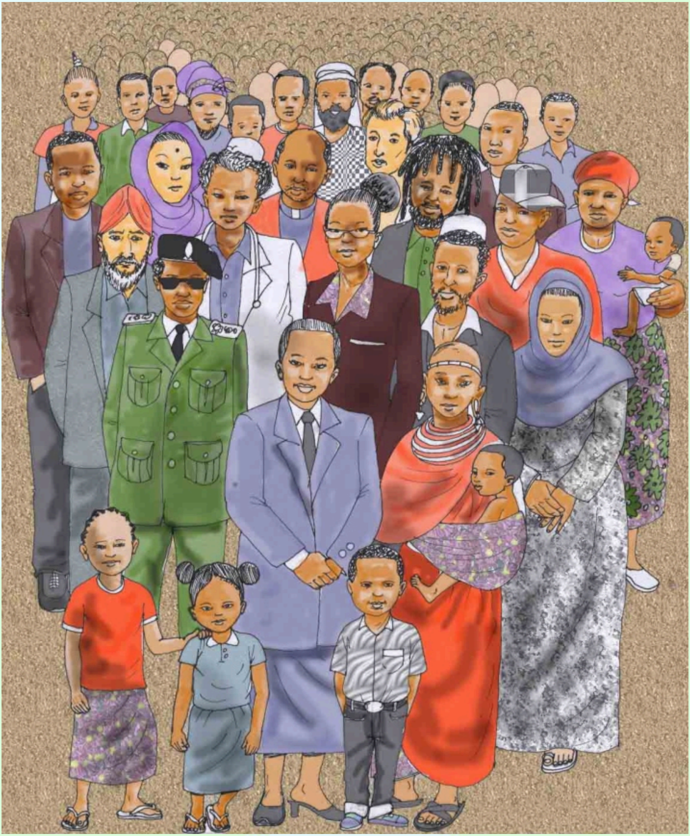

I. My Body

Let's start with an overview of our body. It is not only the doctors who must understand our medical needs. We must understand them too!
Our body is a very complex place where complex processes take place to keep us alive. It is made up of many organs. Each one of them has a specific function and they are all connected together.

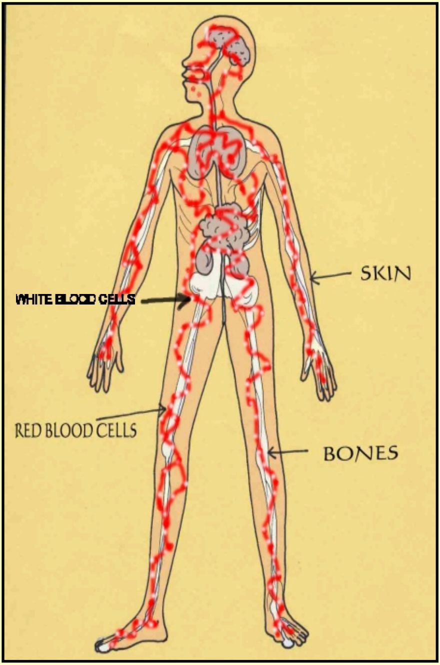

1. The blood

The blood is like a river that flows all over our body.
The blood is a red liquid made up of different cells.

- The **red cells** carry around oxygen to every organ and ensure their survival.

- The **White blood cells** defend our body each time a germ tries to attack it.

2. CD4

- Within the group of white blood cells, there is a very important family:

The Cd4

They are our protector. They are very active when germs that make you sick invade your body. They fight like soldiers during a war. As they are very many of them, they kill the germs.

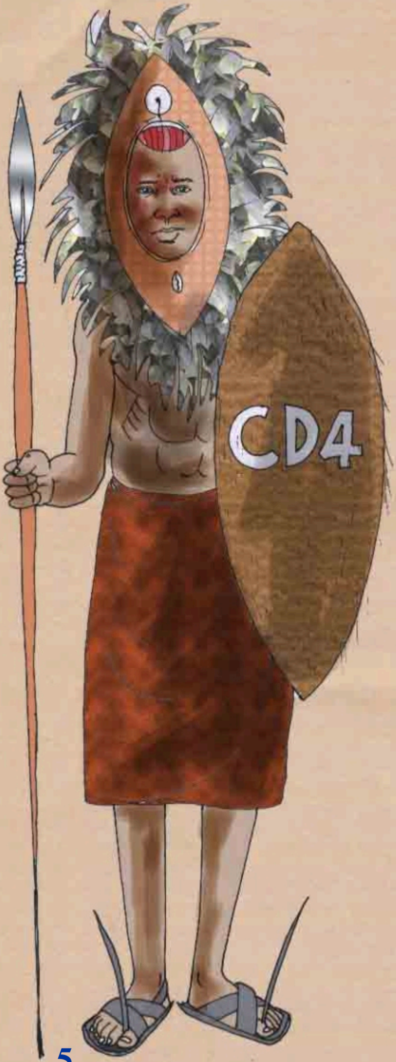

II HIV

1.What is HIV?

HIV means Human Immuno-deficiency Virus; it is the name of the virus that causes AIDS.

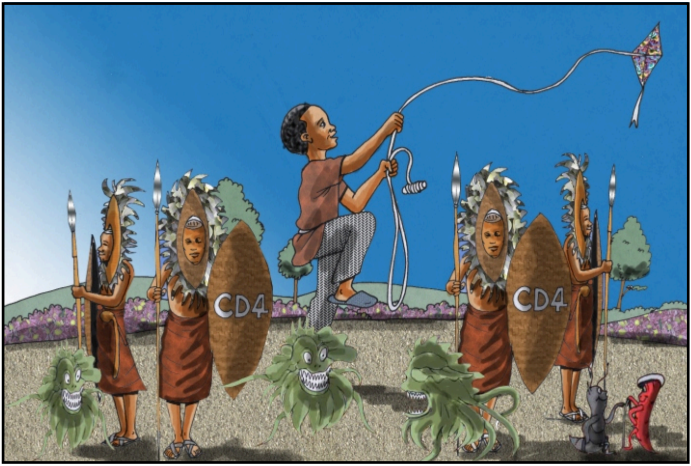

When your body is in good health, your blood circulates without you noticing it and our friends the CD4 watch over your body like guards on everything that could attack it. When the HIV virus enters into your blood, it progressively prevents the CD4 to do their job. But remember, being HIV+ is not being sick. It means that you have the virus in your blood.

2. How does the virus weaken your body?

THE FIRST STEP

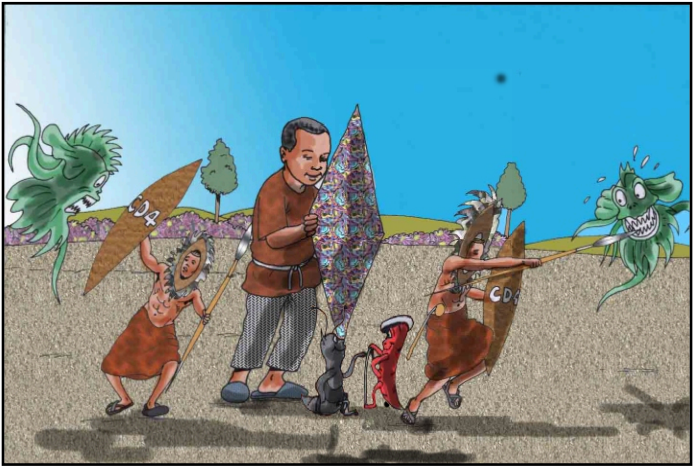

At the beginning the CD4 gather and fight so well that they keep you healthy. You don't notice that the HIV is in your body. You feel good, you are not sick, you are normal and this can last for several years...

THE SECOND STEP

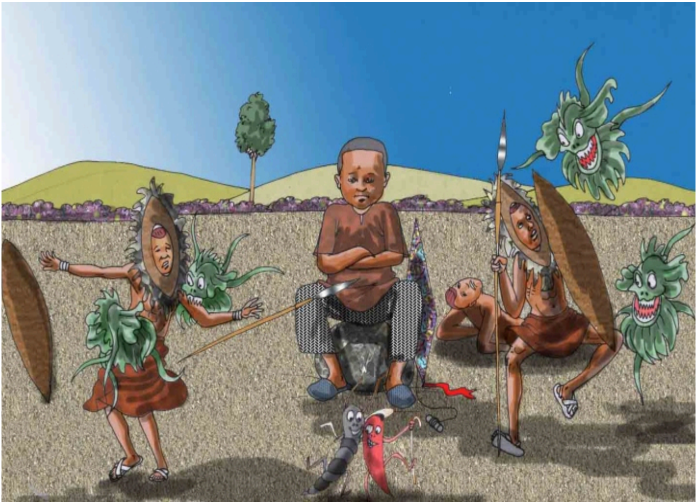

Little by little, the HIV destroys your friends the CD4. But the ones that are still there fight like brave friends.

THE THIRD STEP

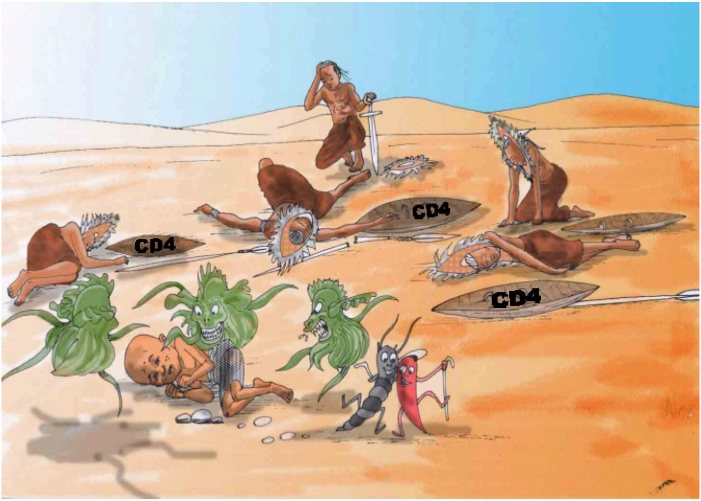

By fighting so hard, the CD4 end up exhausted and too weak to defend your body against diseases.

III. AIDS

THE FOURTH STEP

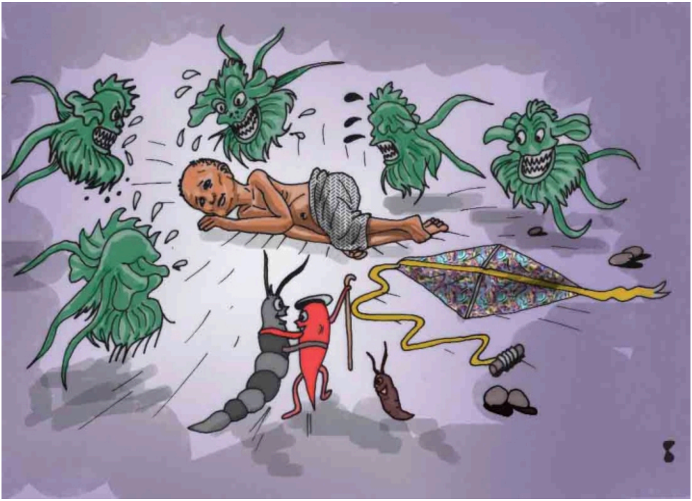

This fourth step is called AIDS

Acquired Immune Deficiency Syndrome.
This is when the HIV virus has weakened your body's defences and germs of all kind are attacking you.

You can develop fever, diarrhoea, headaches…

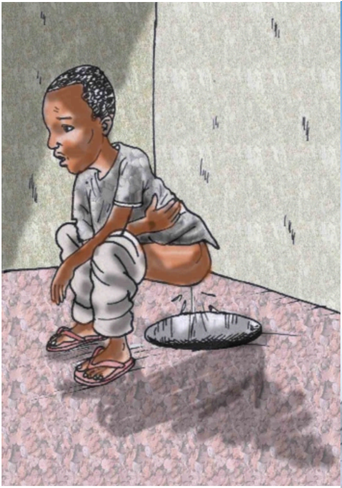

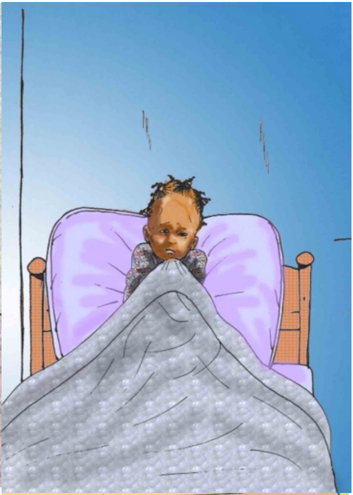

Life becomes difficult. You will become sick more easily with diseases like TB, skin infection, diarrhoeal diseases... These diseases are called Opportunistic Infections (OIs) because they take the opportunity to attack when your body is very
weak. AIDS can develop within 3 to 15 years after you get infected with HIV. However, remember that most of the OIs can be treated successfully!

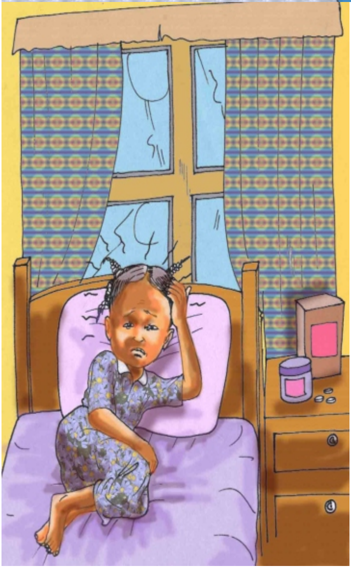

IV How do we get infected by HIV

For children, there are 2 ways of transmission; for adults and youth there are 3.

Children

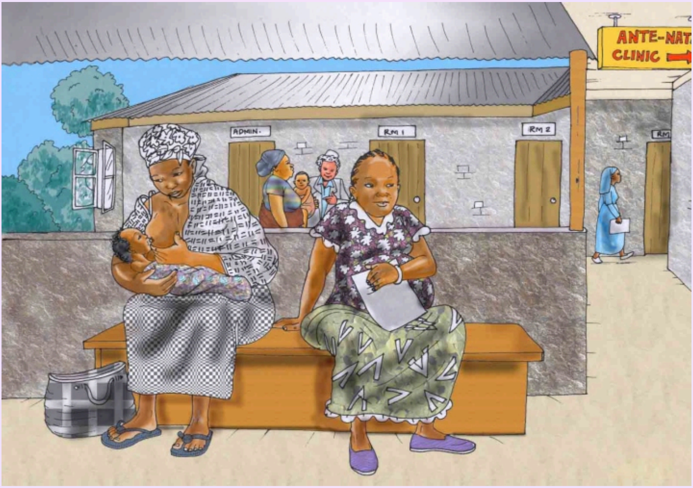

➢ 1st way: Mother to child. If your mother is infected, the virus can infect the baby's body

- When you are in her belly during pregnancy;
- During delivery;
- During breastfeeding.

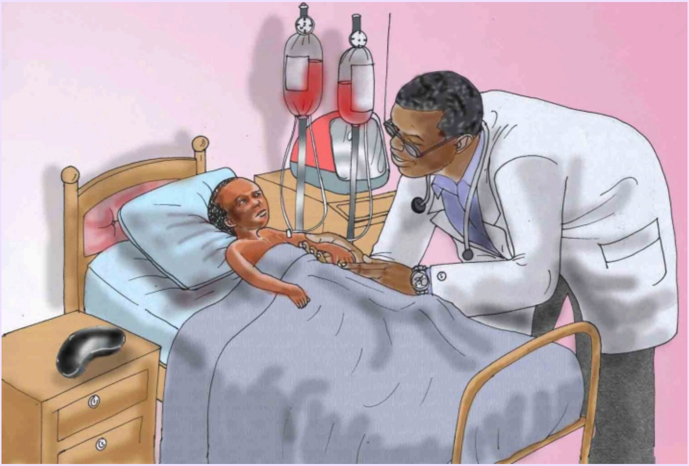

➢ **2nd mode of transmission to children: Contact with blood.**

Each time your are in contact with blood from someone infected with HIV you risk getting infected yourself. For example:

- If you receive a blood transfusion.
- If you touch or cut yourself with a blade already used by someone else (after a man shaved, or cut his hair…).

You have to be very careful and never touch blood or bloody things. Be very careful with all sharp things, knives, blades, scissors…

Adults and young people

In addition to these 2 ways of transmission affecting children, a third way of contamination exists for adults and young people. A woman or a man can get infected when she or he has unprotected sex (without condom)

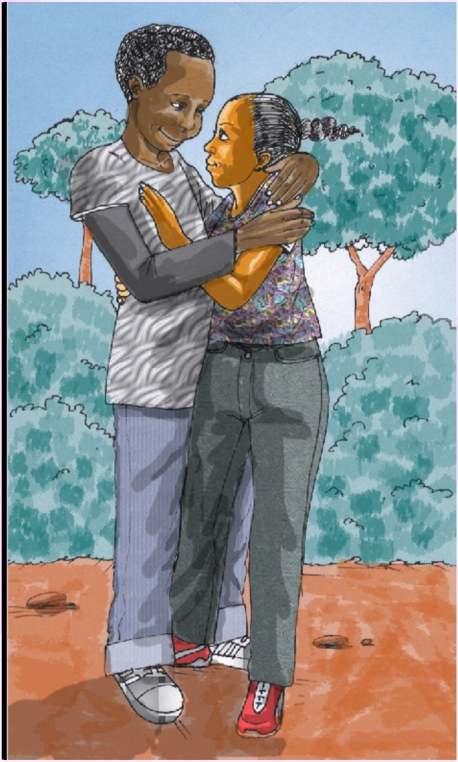

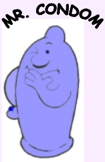

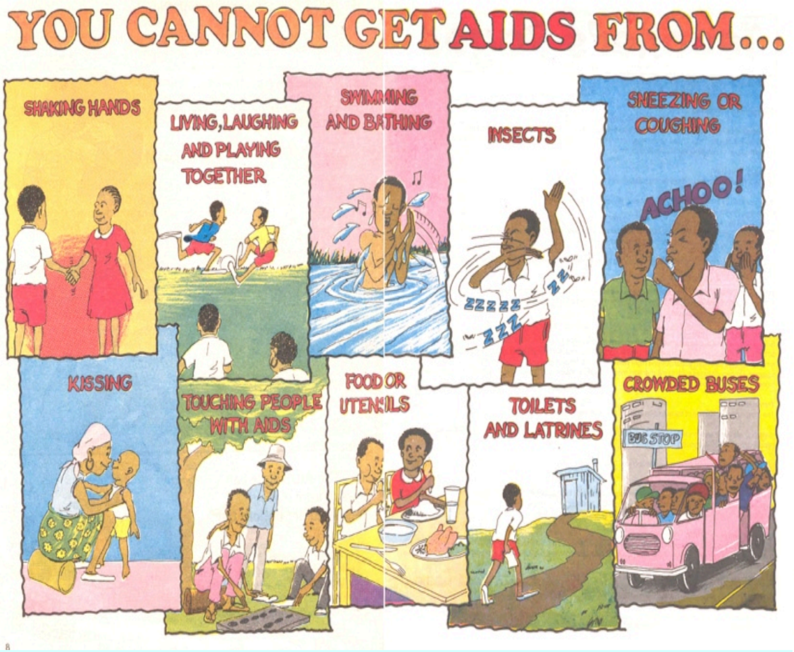

V. Treatment / Drugs ARV, Cotri

1. Why take Cotri?

When your body is weak and in order to prevent diseases, the doctor will prescribe you a drug with a very complicated name called: Cotrimoxazole. Lets call it " cotri", it is easier.You know it also as Septrim. If cotri is prescribed, you have to take it every day !!! It will help your body to avoid Opportunistic Infection like lung infections, diarrhoea,...

2. What is the CD4 count?

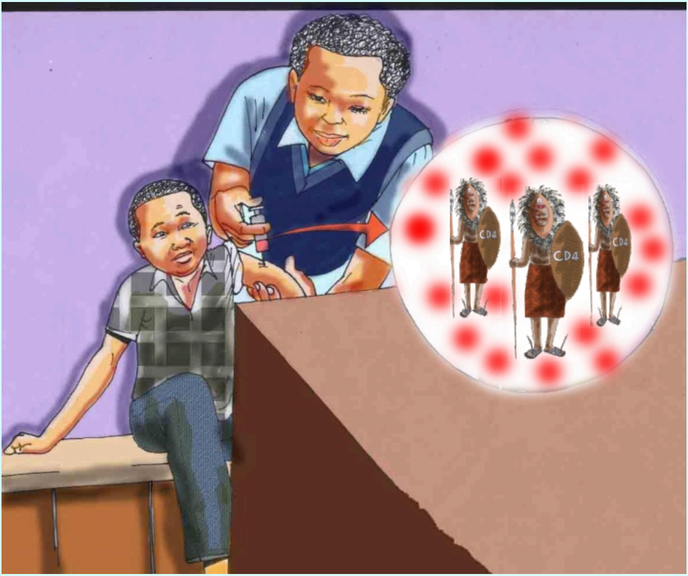

At the same time as prescribing "cotri", the doctor will ask you to take a lab exam: the CD4 count. This is to see how many of your friends the CD4 are present in your blood. If there are enough, you continue the preventive treatment with cotri. If they are not enough, he'll propose that you start ARVs.

3. What are the ARVs ?

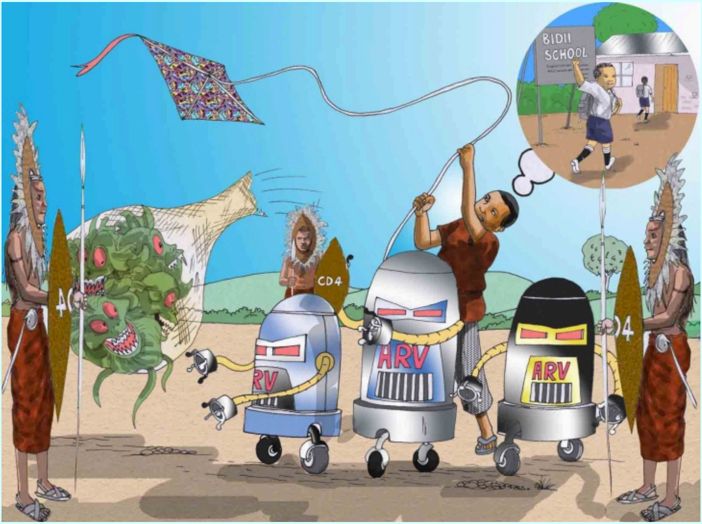

Antiretroviral (ARVs) are your friends. They don't eradicate the virus from your body but they are the only medicine that can stop the HIV virus from multiplying in your body. ARVs helps the precious CD4 to get strong so that they can keep away Opportunistic Infections and thus give your body a chance to become strong again. After few month, if you sleep well, eat well, gain weight, continue to do sport and meet your friends, you will feel strong again, you will feel full of energy. You are not sick anymore and you can return to school or to work, you can play games and go back to your normal life...

The public perception that "there is no cure for HIV" is confusing. It is true that at the moment, there is no cure for HIV. However there is also no cure for potentially deadly chronic illnesses such as diabetes or high blood pressure. Nevertheless, people who have these illnesses can live normal lives provided they stay on treatment.

NOTE for young people and adults: use a condom every time when having sex because even while taking the ARVs, one can still transmit the virus and/or get re-infected with a different strain of HIV that might interfere with the treatment.

MR. CONDOM

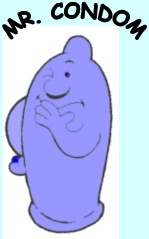

&

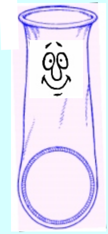

MRS. CONDOM

4. 3 conditions for ARVs to stay your good friends

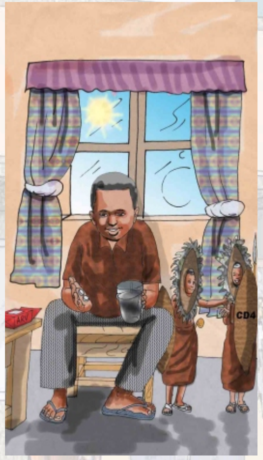

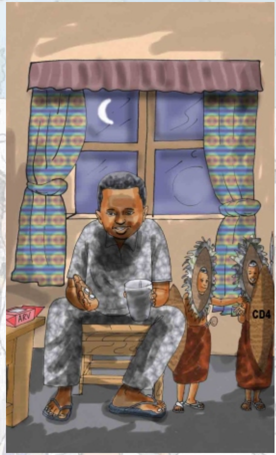

- You must take your ARVs every day, morning and evening at the same hour.
- Don't forget them, otherwise the virus will find tricks and struggle so well that ARVs will not be able to save you anymore.
- You should know that until today, ARVs are a life-long treatment. Once you have started you should be monitored by your health care provider. But let's hope that scientists will find a vaccine and a treatment against HIV. In the meantime, be reassured. If you take them correctly, ARVs will stay your faithful friends.

THANKS ARVs

5. ARV drugs might not work if:

- You drink alcohol
- You take herbal therapy
- You practice unprotected sex
- You do not observe good nutrition
- You do not treat the OIs as they occur
- You don't take ARV every morning and every night at the same hour

NOTE: It is important to always tell your doctor the truth about your medication or anything you have (fever, skin rash...)

6. What are the ARV Regiments?

You will be treated with three ARV drugs combined in 1 pill. This combination reduces the number of pills to take per day. The drug combinations and number of pills can be different for children or if you are being treated for TB or if one ARV doesn't suit you. If the standard regiment doesn't suit you, you can benefit from another combination of 3 different ARVs, which we call "second line" treatment.

7. ARV Side Effects?

As is the case with any other medicine (e.g. "cotri"), ARV drugs have side effects. Side effects are different from drugs not working. REMEMBER that some people have side effects and some people don't. It is not possible to predict who will get them and who will not. Most side effects occur during the first few months after beginning ARV treatment, but some can take a long time to develop. Being sick from the virus is worse than making it through the first few weeks of side effects. Side effects usually go away after your body gets used to ARV drugs. If they don't, you may need to try another drug. You can have nausea, vomiting, mild diarrhoea, abdominal bloating, headache, mild fever, itching of the body, lack of sleep, too much sleep, nightmares, skin rash (mild to severe)...

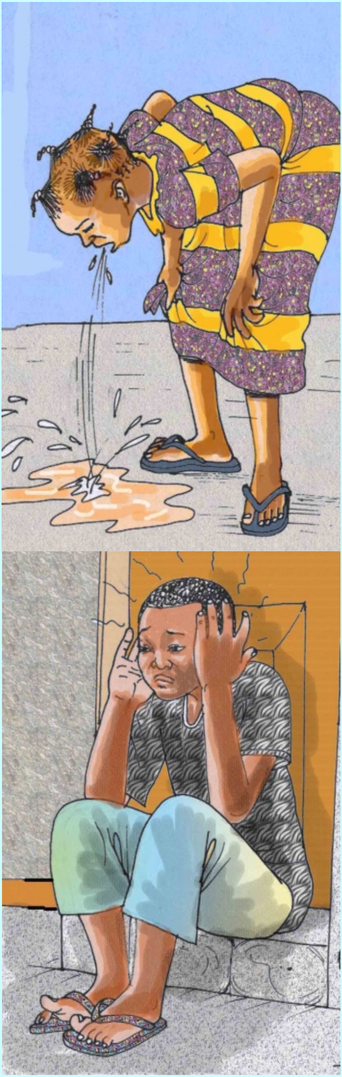

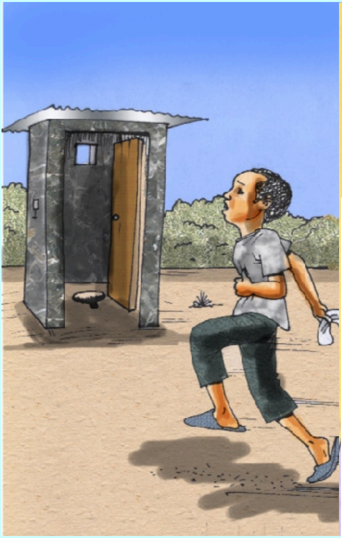

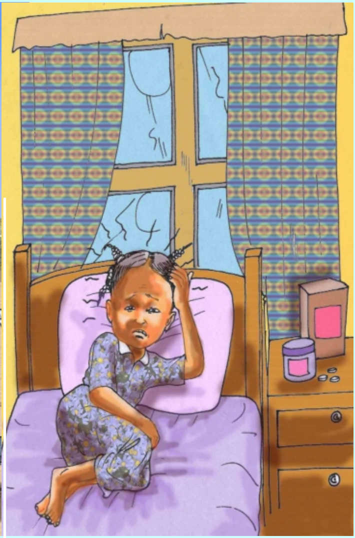

The majority of these side effects will disappear progressively but if they don't, always inform your caregivers or your treatment assistant and VISIT YOUR DOCTOR as soon as possible. You will be followed up.

Safety

Some other drugs may decrease the effectiveness of ARV drugs. If you are being given other medication, discuss this with your doctor. Also, if you attend another hospital, inform them about the ARV drugs you are taking.

VI. Nutrition

Good nutrition includes adequate and optimal amounts of carbohydrates, proteins, fats, vitamins and minerals. These should constitute a well balanced diet. Try to eat food from each of these groups every day.

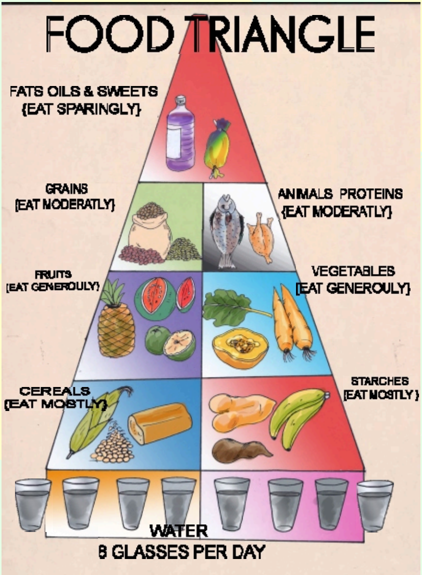

What is a well balanced diet ?

Body-building food (protein) : beans, soya, eggs, meat, fish, chicken.

Energy-giving foods : maize, millet, rice , potatoes, sugar, oils and fats.

Food with vitamins that protect against infections : fruits and vegetables.

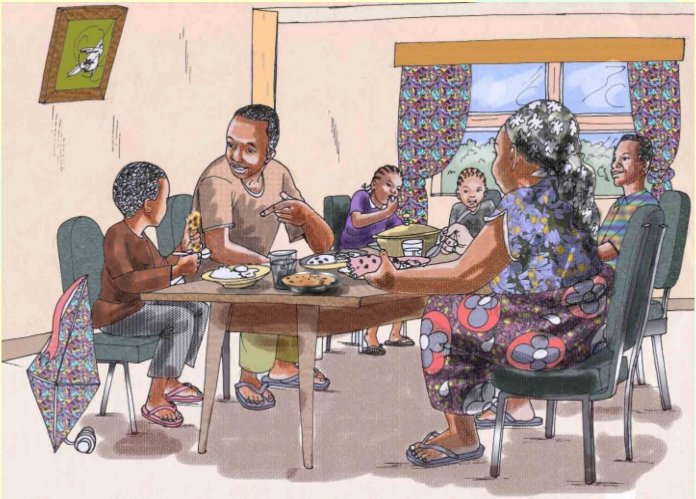

Nutrition advises:

Remember:

- There is no special food for HIV positive persons; you just have to eat normally along with your family and friends.

- Drink plenty of water every day

- Balance your diet according to the food triangle

Also a healthy diet should be part of a lifestyle that includes:

- Exercise
- Fresh Air

VII. Personal Hygiene

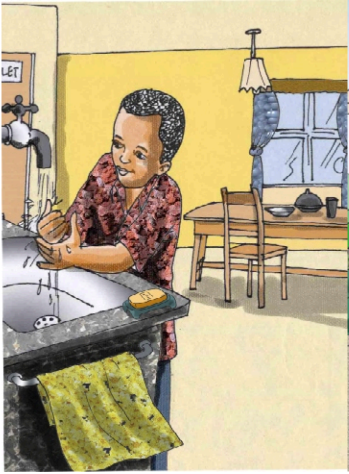

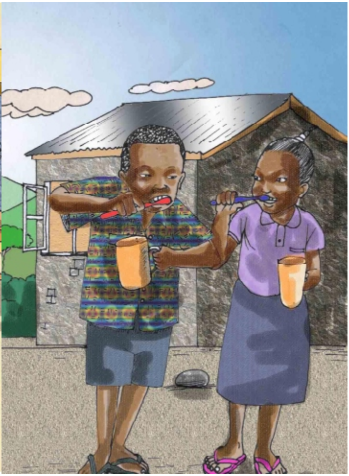

These are advices to keep a healthy life. To feel good with your body and mind:

- Wash your hands before and after going to the toilet.
- Wash your hands before cooking, before and after eating.
- Brush your teeth in the morning and before going to bed.
- Keep your nails short and clean.
- If you buy food prepare outside your home, make sure it is clean and well cooked.
- Try to bath at least twice a day.
- Wear fresh, dry and clean underwear, change them every day.

Girls: Remember to change your sanitary towel frequently.

Boys: Change your underwear daily even if it doesn't look dirty.

VIII. Disclosure

It is very important to disclose, to reveal your status to someone you trust. She or he can be an important support to you. BUT remember it is important to know:

- To WHOM to disclose
- WHEN to disclose
- and WHY to disclose.

IX Confidentiality

Your medical data will be identified by a number and will be analysed along with information about other patients. This information may be published, but your name and your address will never ever be published.

Your parents or caregivers are not giving up any of your legal rights by signing the consent form.

After you have read the booklet entirely, please feel comfortable to ask as many questions as you have to our staff.

If you are not selected to be on ARV drugs, good medical and laboratory monitoring is still important. You will be scheduled for regular medical visits and CD4 cell counts.

Selection for taking ARV drugs will be reviewed later.

X POSITIVE LIVING

LIVE A POSITIVE LIFE AND REMEMBER :

- Keep playing
- Make friends
- Keep yourself busy,
- Being HIV+ doesn't mean you are sick
- OIs can be prevented and treated
- Take your tablets at the same time every day
- Honour all your clinic appointments and always tell your doctor the truth
- Talk to someone you trust in case you are worried
- Protect yourself

"You must be the change you wish to see in the world" *Gandhi*

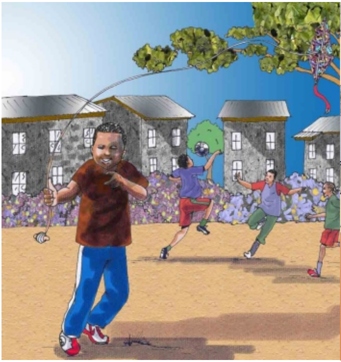

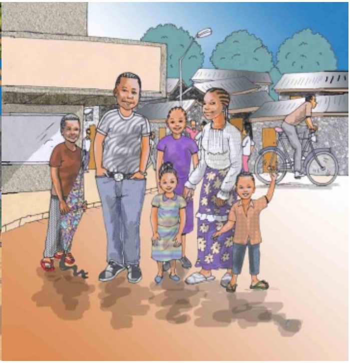

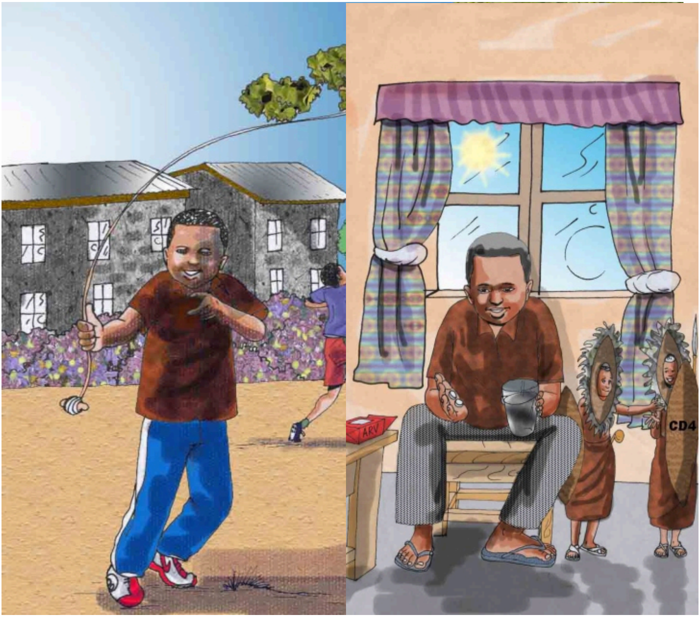

Being HIV+ doesn't mean you are sick

Most of the OIs can be treated successfully!

NEVER FORGET TO LIVE A POSITIVE LIFE!

Based on Suzanne Hervier text

Adapted: Zaina Ahmed, Angela Mutonga, Dorcas Kerubo, Augustine Mwenga,Van Winghem Joelle and Monique Wajanla.

Illustrator: Alfred Ombati

MEDECINS SANS FRONTIERES
P.O. Box 38897 - 00623 NAIROBI
Tel: (254) 2 3870021/25
Fax: (254) 2 3872157 / 3873314
