# Supplementary material for: Implementation of a comprehensive program including psycho-social and treatment literacy activities to improve adherence to HIV care and treatment for a pediatric population in Kenya
Source: BMC Pediatr. 2008 Nov 21;8:52. doi: 10.1186/1471-2431-8-52 (PMC2613143; doi:10.1186/1471-2431-8-52)
Supplement: Additional file 2 — "All you need to know about HIV and ARVS Youth Booklet". a pocket size booklet containing basic information about HIV, AIDS, opportunistic infections (OIs), HAART, nutrition and positive living attitudes for teenagers. [file 1471-2431-8-52-S2.zip › Thanks%20ARVs.pdf]

THANKS ARVs

A Fairytale that Encourages General Well Being

AUTHOR: NISACHOL OUNJIT
ILLUSTRATOR: CELESTINE WAMIRU

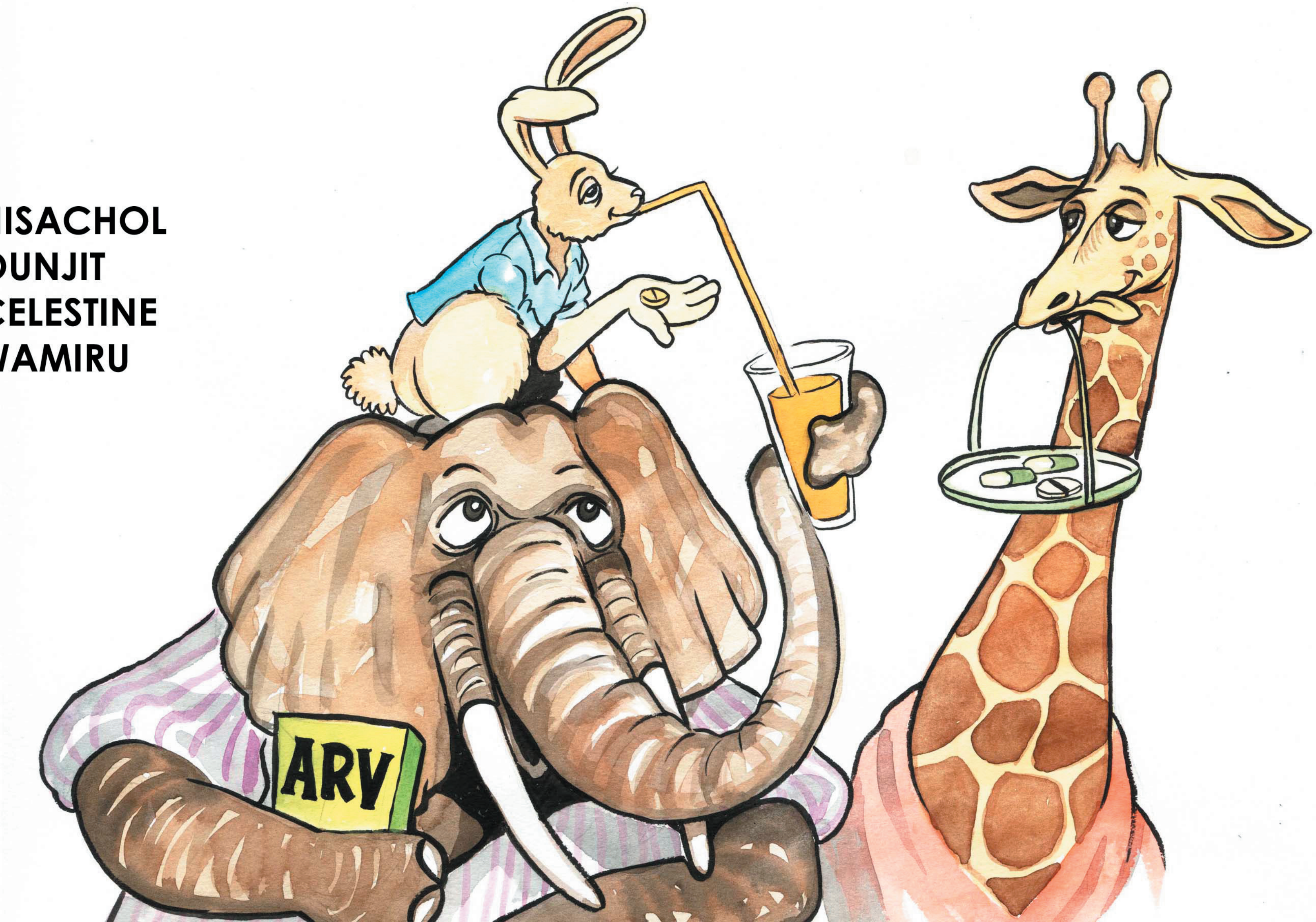

MEDECINS SANS FRONTIERES

August 2006

Introduction:

This fairy tale is written as a communication tool for use with children living with HIV and is aimed at creating a positive understanding of HIV. The fairy tale helps children understand basic concepts about disease and how to keep themselves healthy.

At the end of this book are some questions to assist you in talking with children about their health and psycho-social problems, which also allow the children to express their feelings. When using the questions at the end of this book, please select appropriate questions for use with the children. This fairy tale can be read to children more than once and different questions can be used each time.

In a clean and beautiful village, all the animals lived together happily.

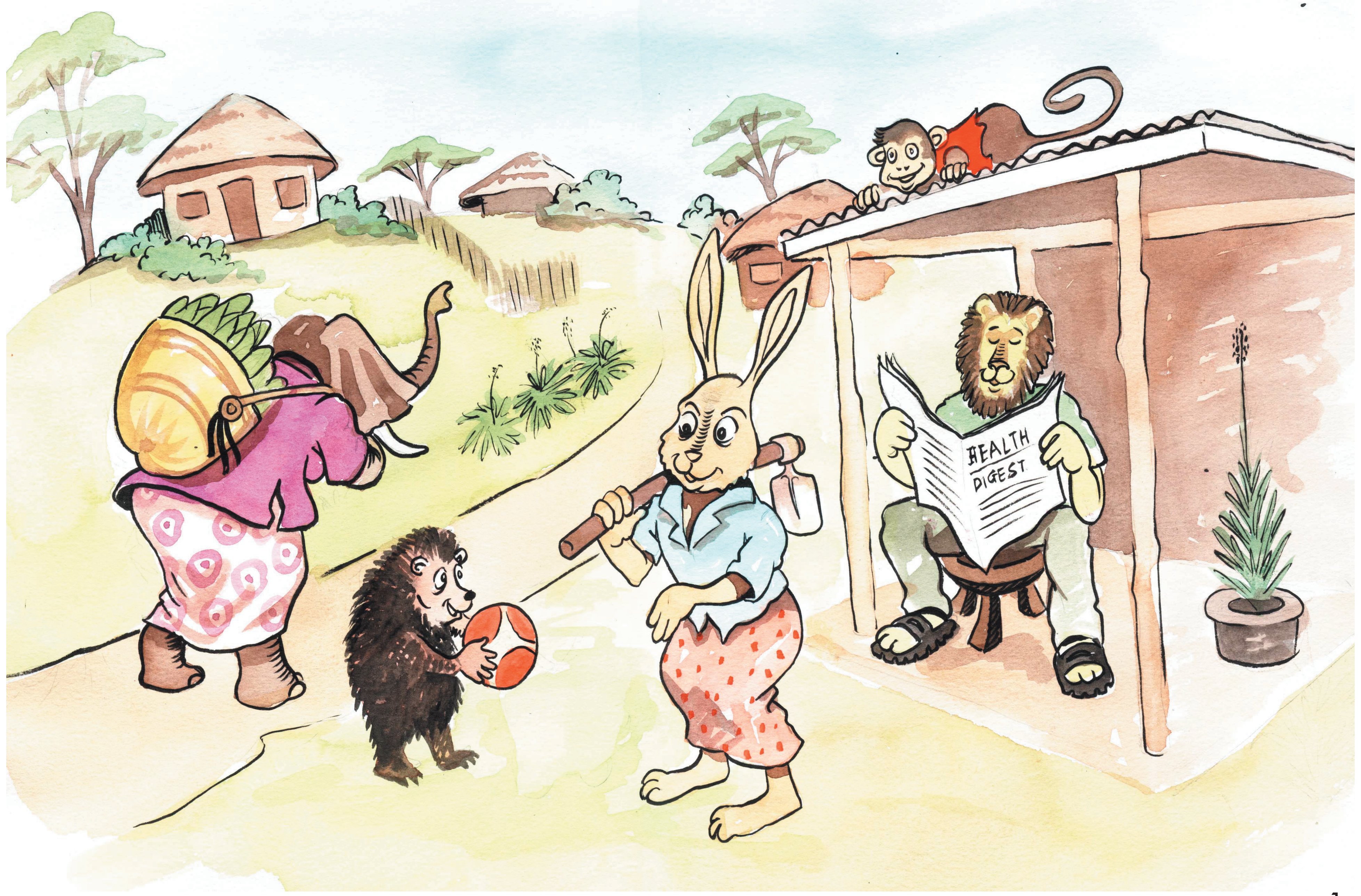

Not far from the village lived a nasty Hyena. She envied the animals and wanted to take over the village. The Hyena decided to try to make everyone in the village ill so that they would die. She put some germs into a bag and flew over the village, spreading the germs on the houses below. The germs started to attack house after house. Little puppy caught flu and had a runny nose. Auntie Elephant got a fever, and little monkey got diarrhoea.

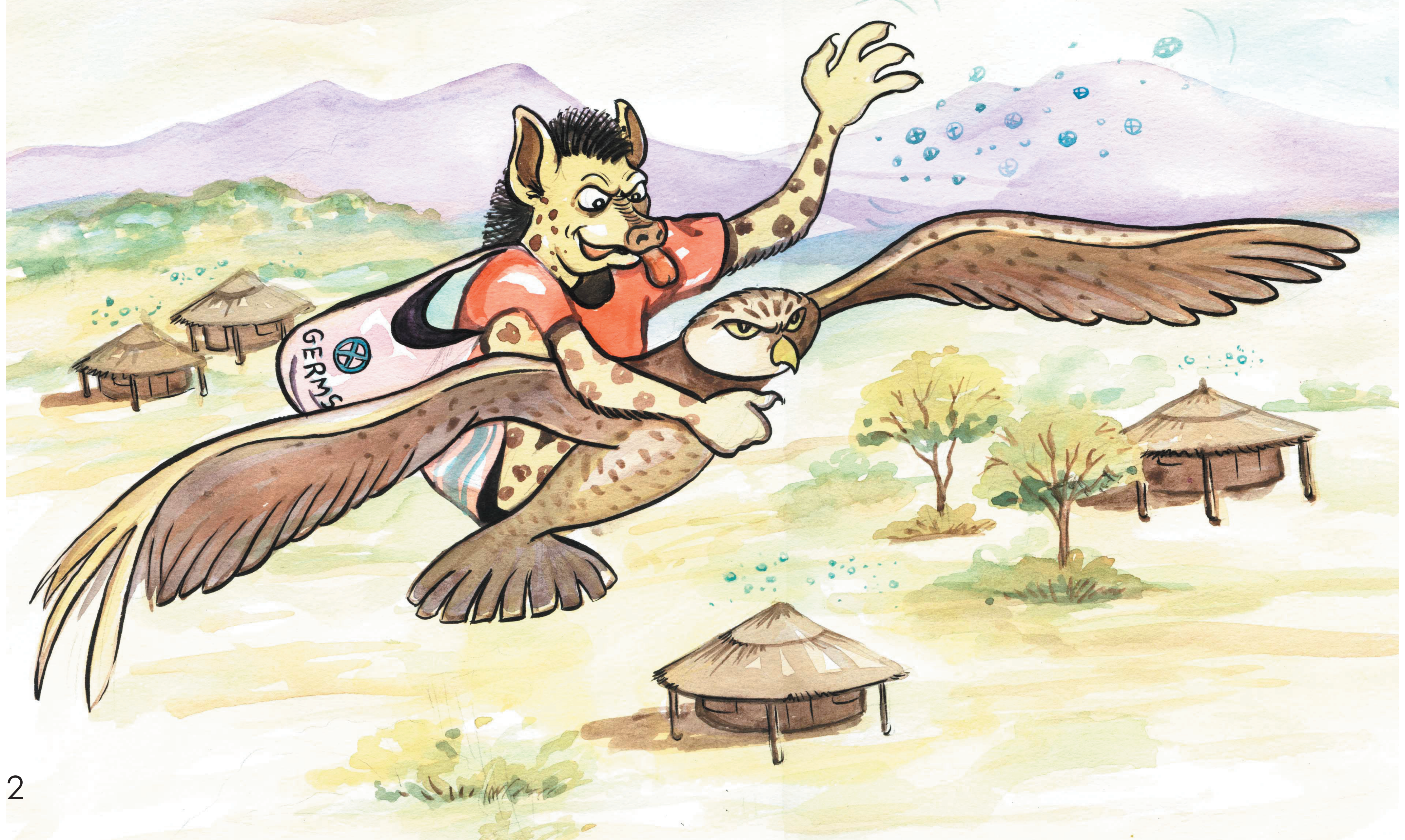

Uncle Lion, the head of the village, reported what was happening in the newspaper and on the radio and TV. He wanted everyone to watch out for the germs that were making many of the young and old sick. Uncle Lion told all the animals to keep themselves healthy and to eat properly so that their bodies could build up a magic force to fight the germs that were attacking them.

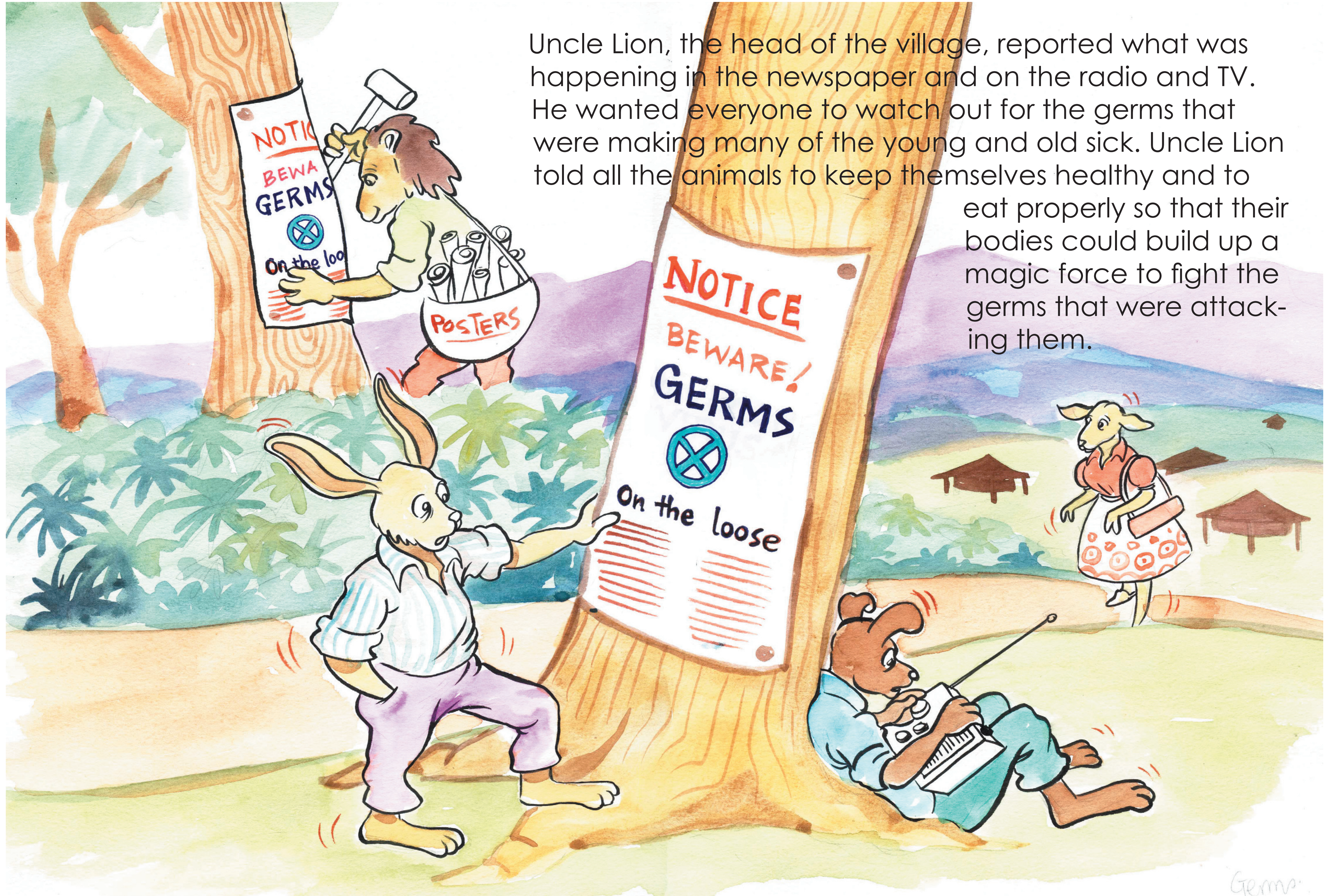

Uncle Lion said that if we eat good food such as rice, meat, vegetables, and fruit; drink milk and clean water; and get plenty of sleep, it will help our bodies to create a magic force to keep us healthy.

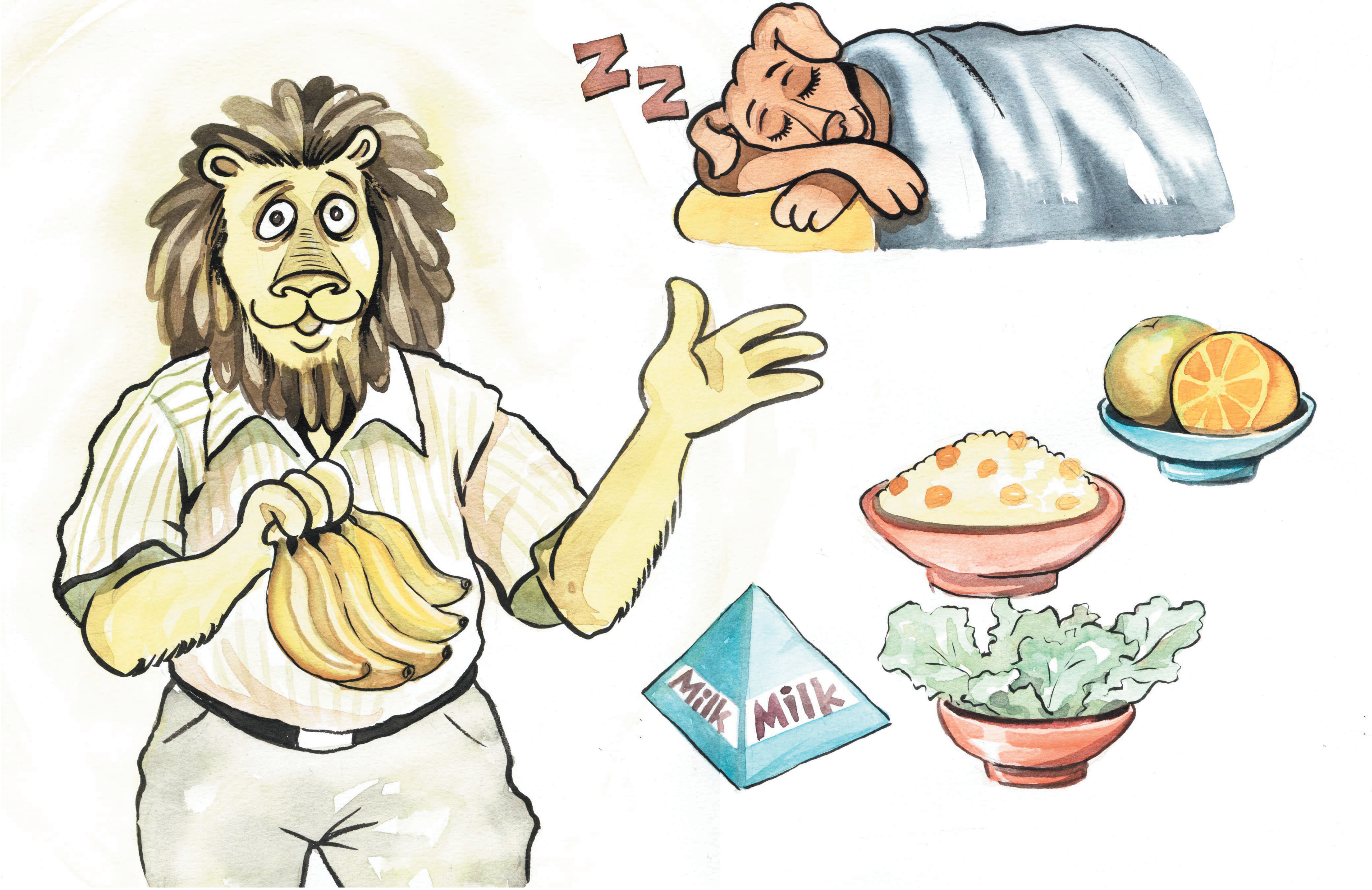

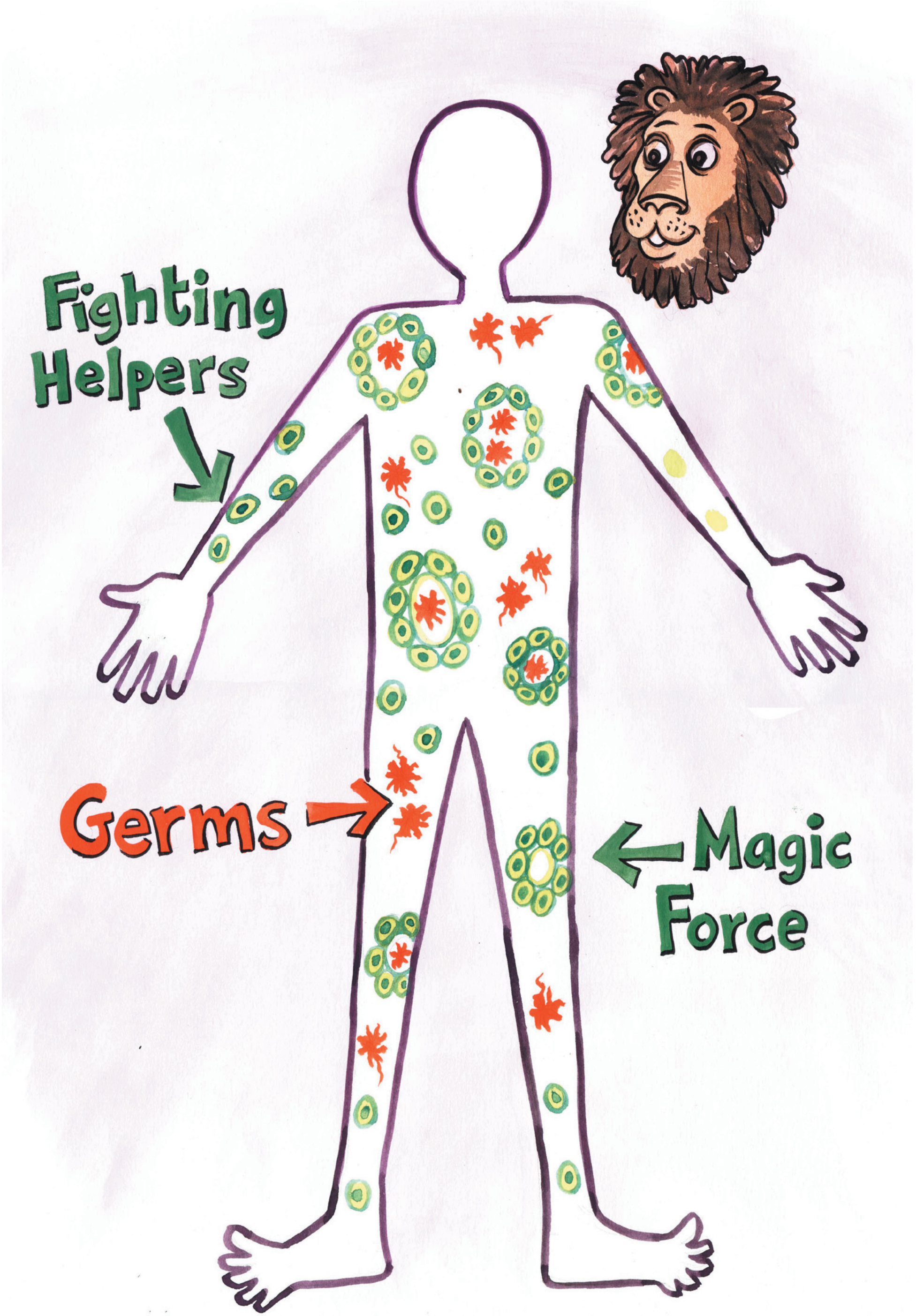

Uncle Lion drew a picture of the body that explained how the magic force can fight the germs that cause diseases. The magic force has assistants called Fighting Helpers. When germs get into our bodies, the magic force calls on the Fighting Helps to attack the germs and stop them causing a disease.

Uncle Lion gave the example of a little puppy who got flu with a cough, fever and runny nose. The magic force attacked the germs that caused the flu and in a few days the little puppy was healthy again.

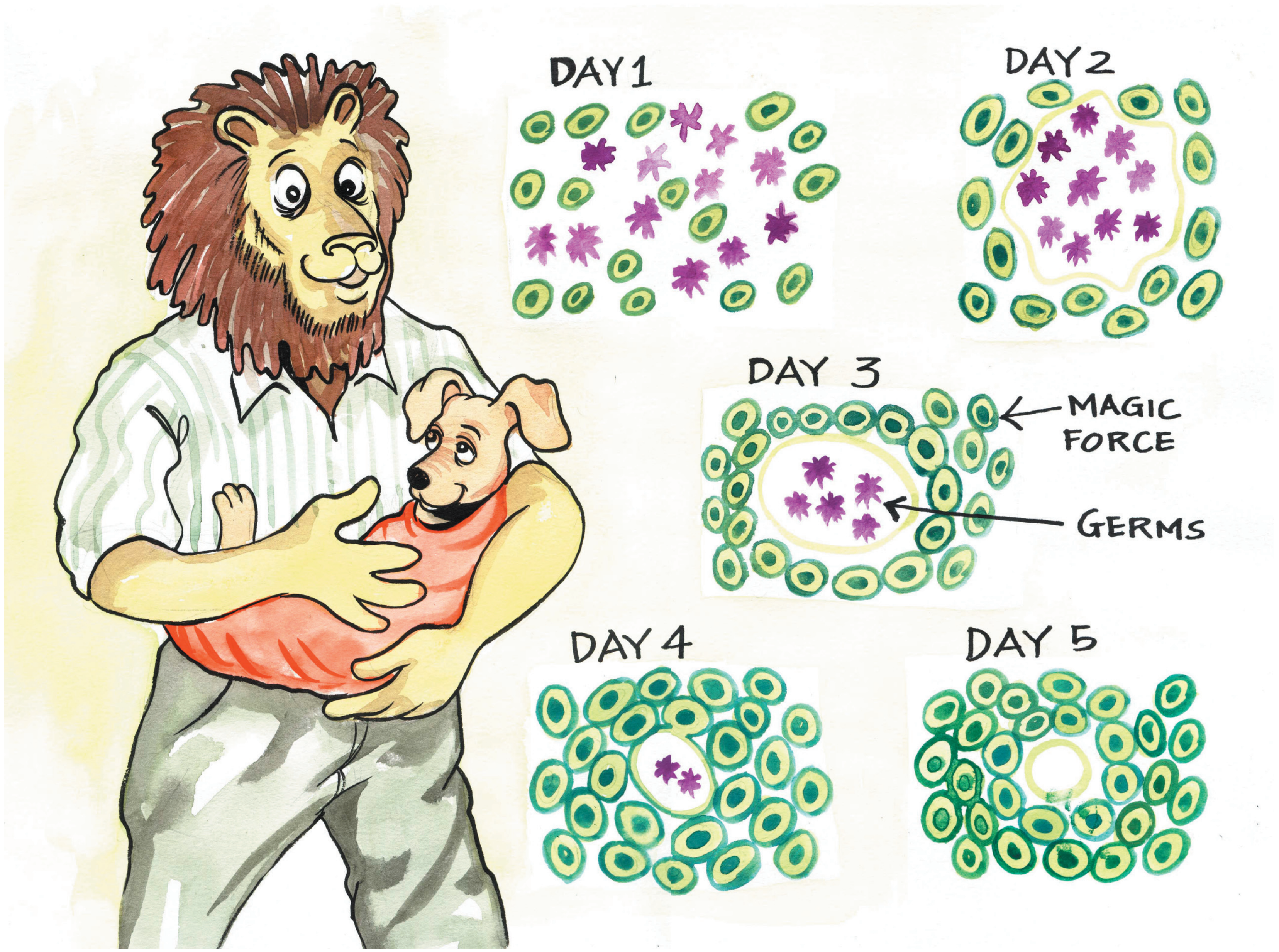

All the animals followed Uncle Lion's advice and built up their magic force. The village was happy again because everyone was strong and healthy.

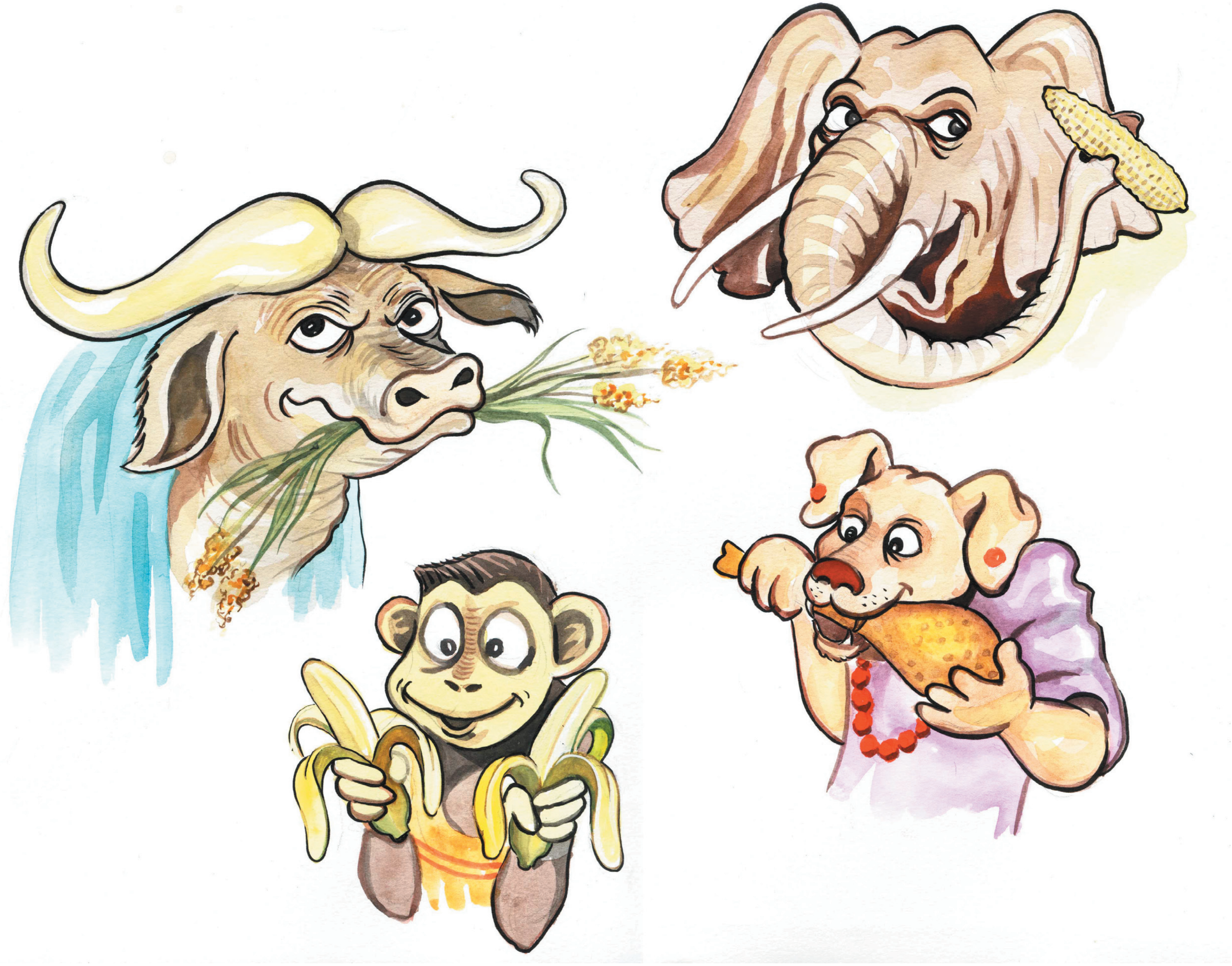

The wicked Hyena never stopped trying to take over the village. She was angry with the germs because they were not strong enough to fight the magic force. The Hyena asked the germs if there was another germ that would be strong enough to take over the village. The germs told the Hyena about a very powerful germ called the Devimon Virus that would be able to beat the magic force.

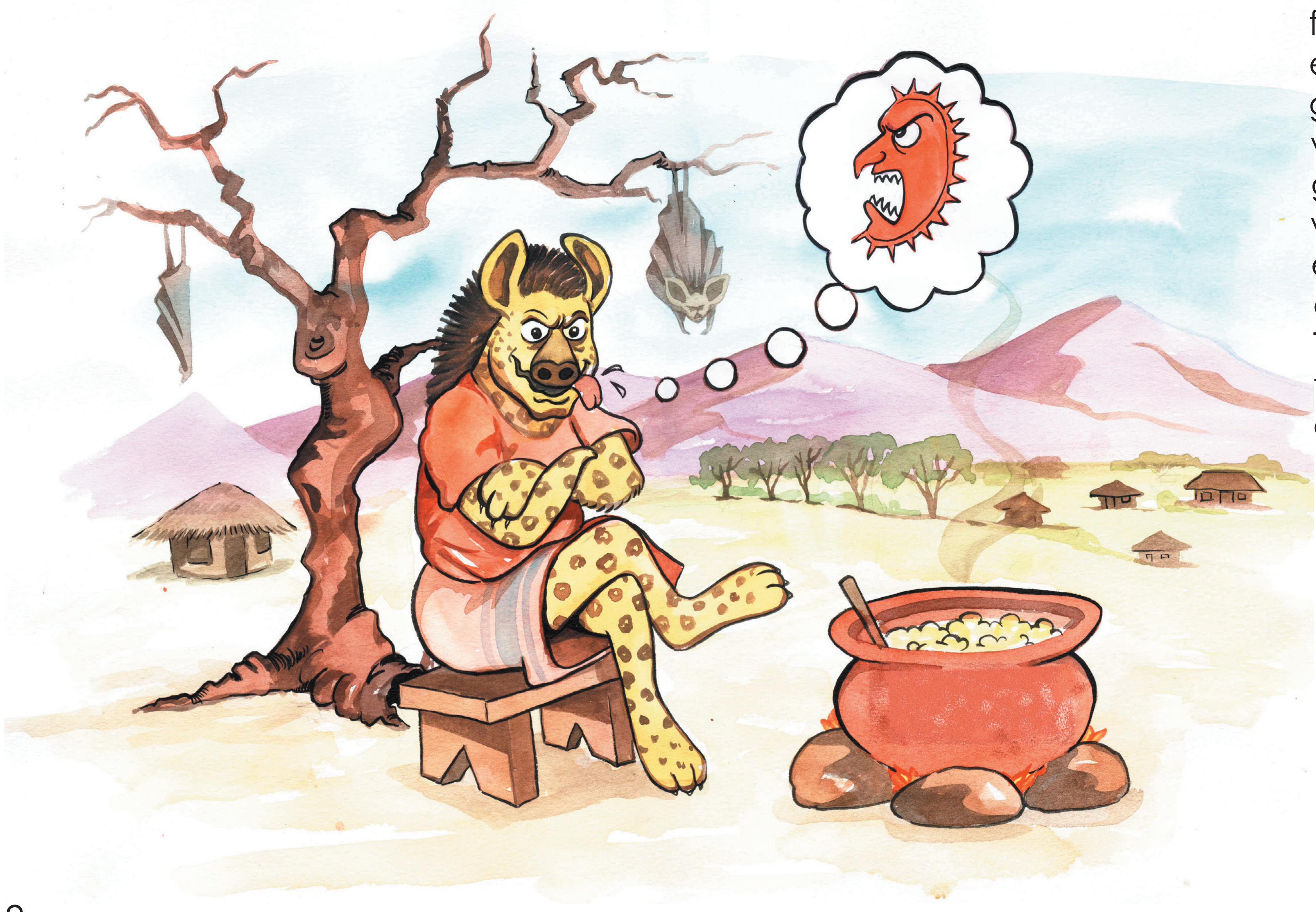

When she heard this, the nasty Hyena flew off to meet the Devimon Virus.The Devimon Virus is a special germ with a round body covered in studs. There are no ordinary medicines that can defeat the Devimon Virus. If the Devimon Virus gets into our bodies it will destroy the magic force and we will die.

When the nasty Hyena met with the Devimon Virus, she asked them to follow her back to the village.

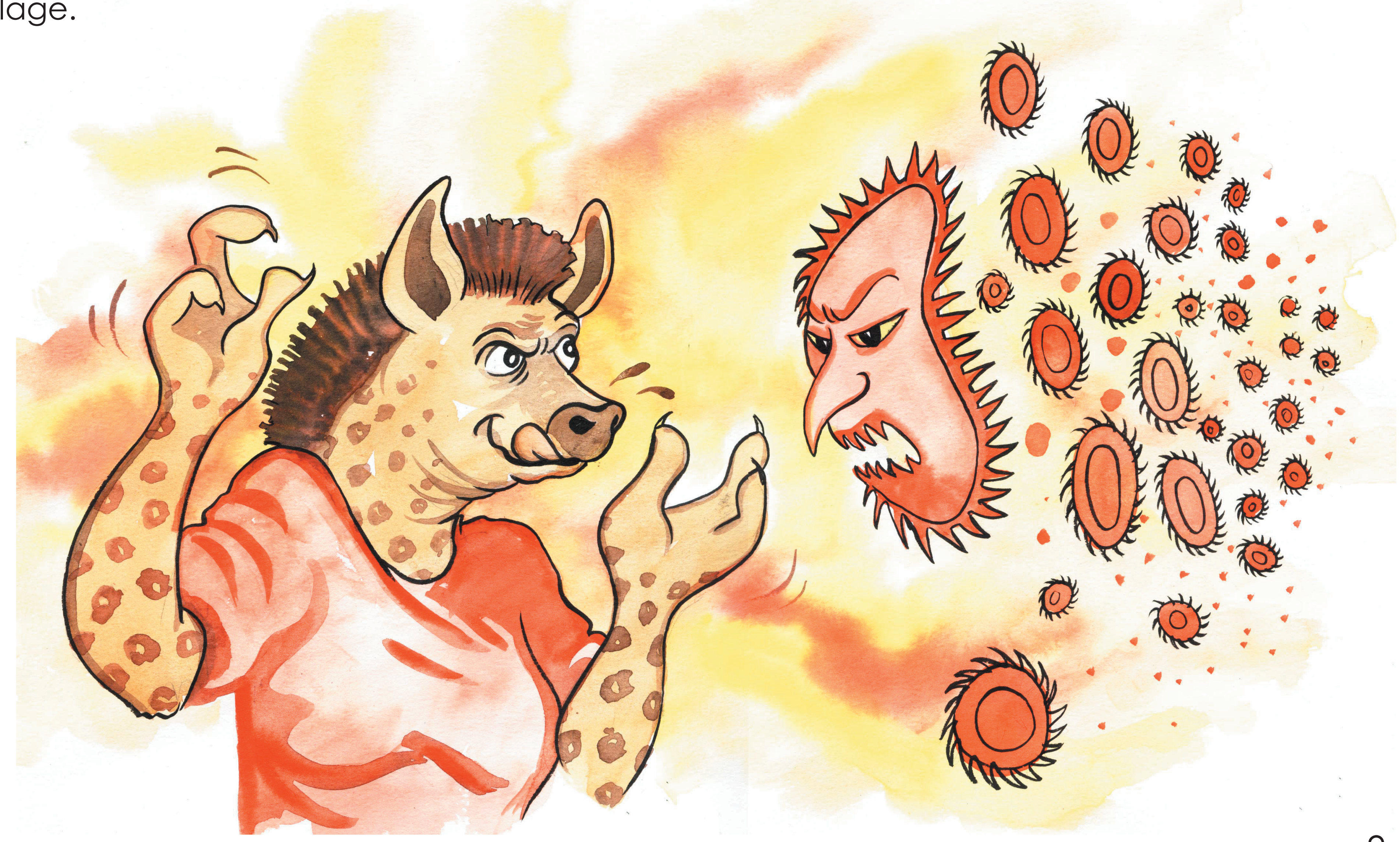

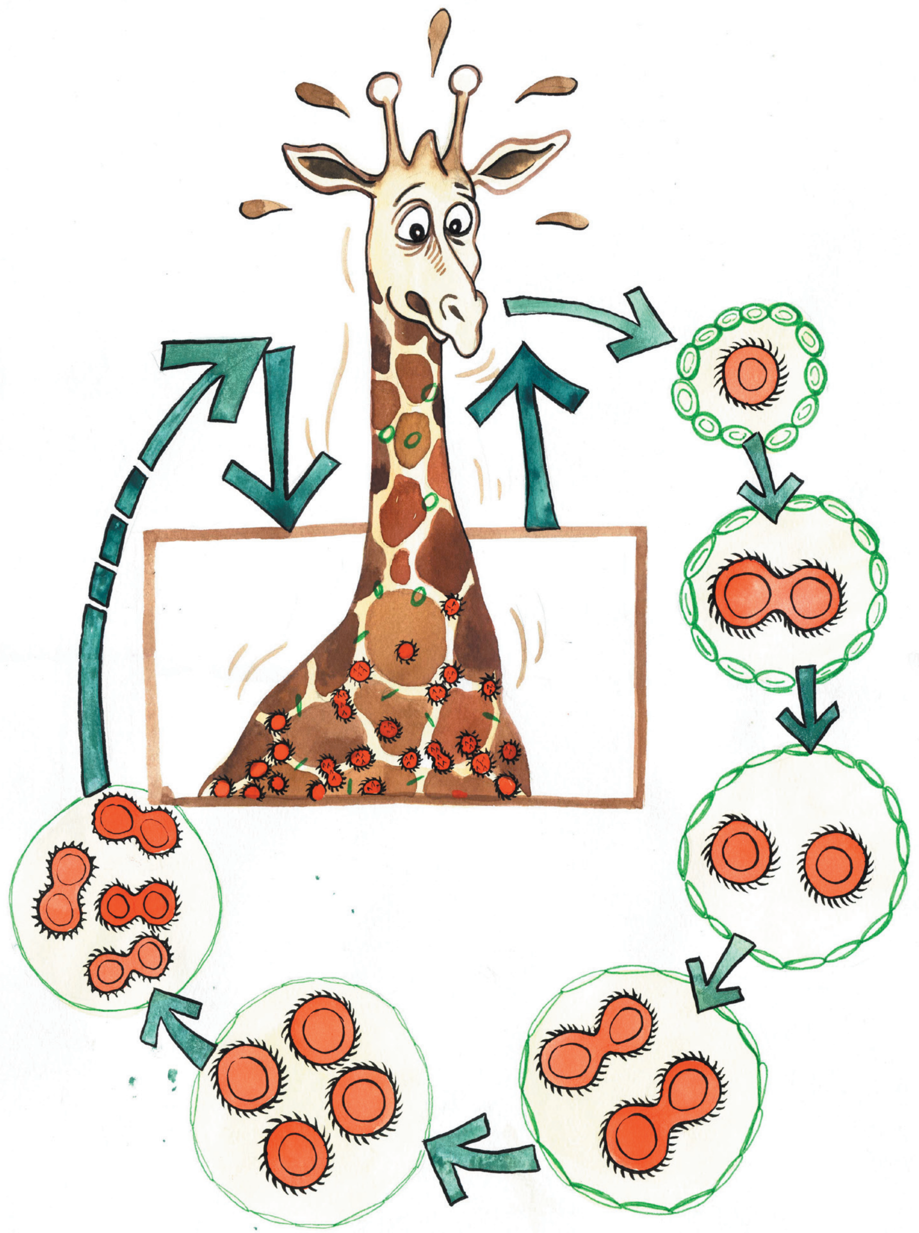

The fighting between the Devimon Virus and the animals in the village went on for a very long time. Uncle Lion told everyone to eat properly and to take ordinary medicines to fight the Devimon Virus. The Devimon Virus used its studs to catch and get inside the Magic Force to make more and more Devimon Virus until the animals were full of Devimon Virus.

The animals started to get sick and soon they began to die.

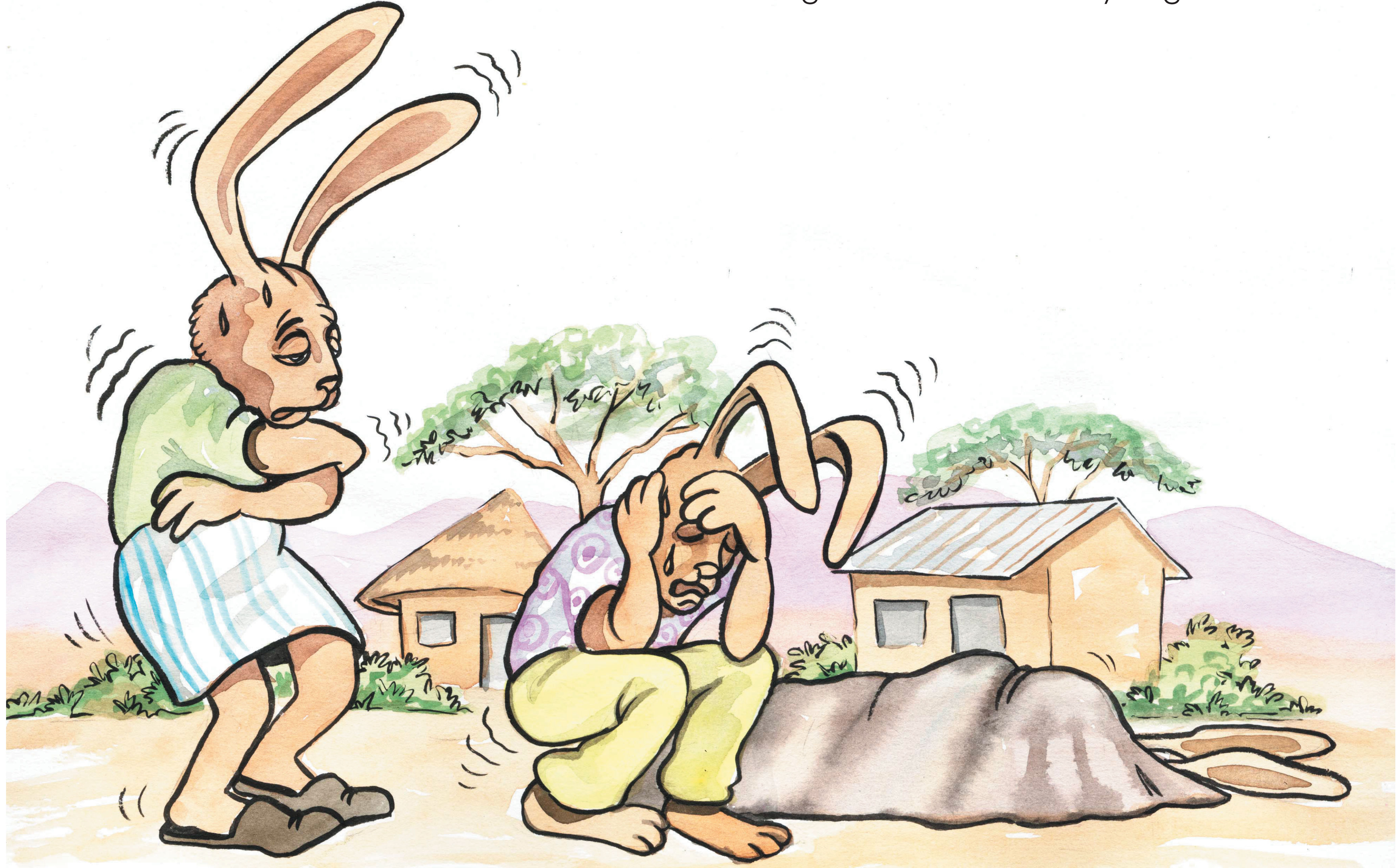

Just eating properly and taking ordinary medicines could not defeat the Devimon Virus so the whole village was very sad but Uncle Lion and Auntie Elephant never gave up. They worked hard and finally discovered a medicine called ARV. Although ARV could not kill the Devimon Virus, it could stop the Devimon Virus from destroying the Magic Force.

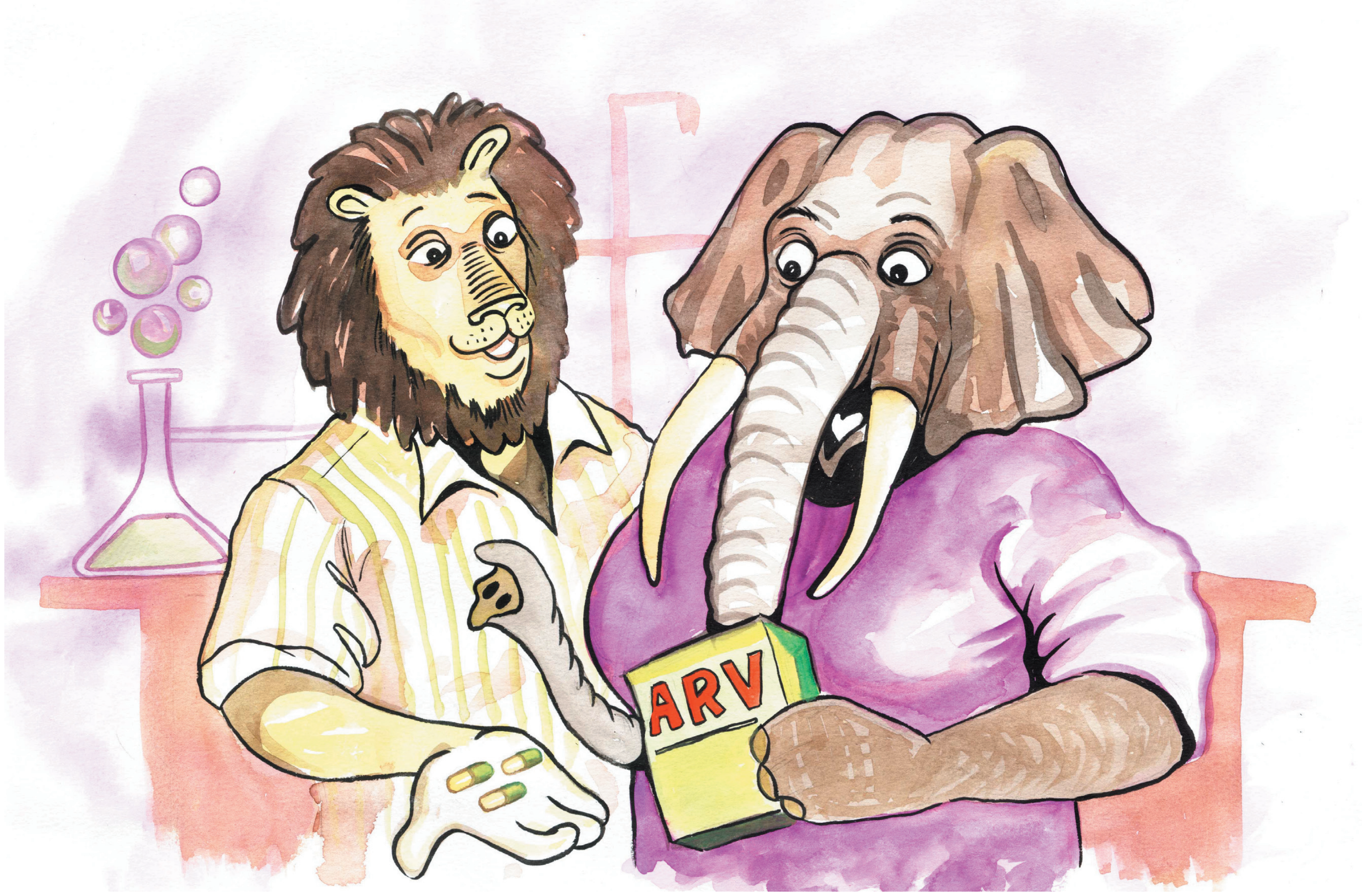

The ARV medicine was made very quickly and given to all the animals in the village.

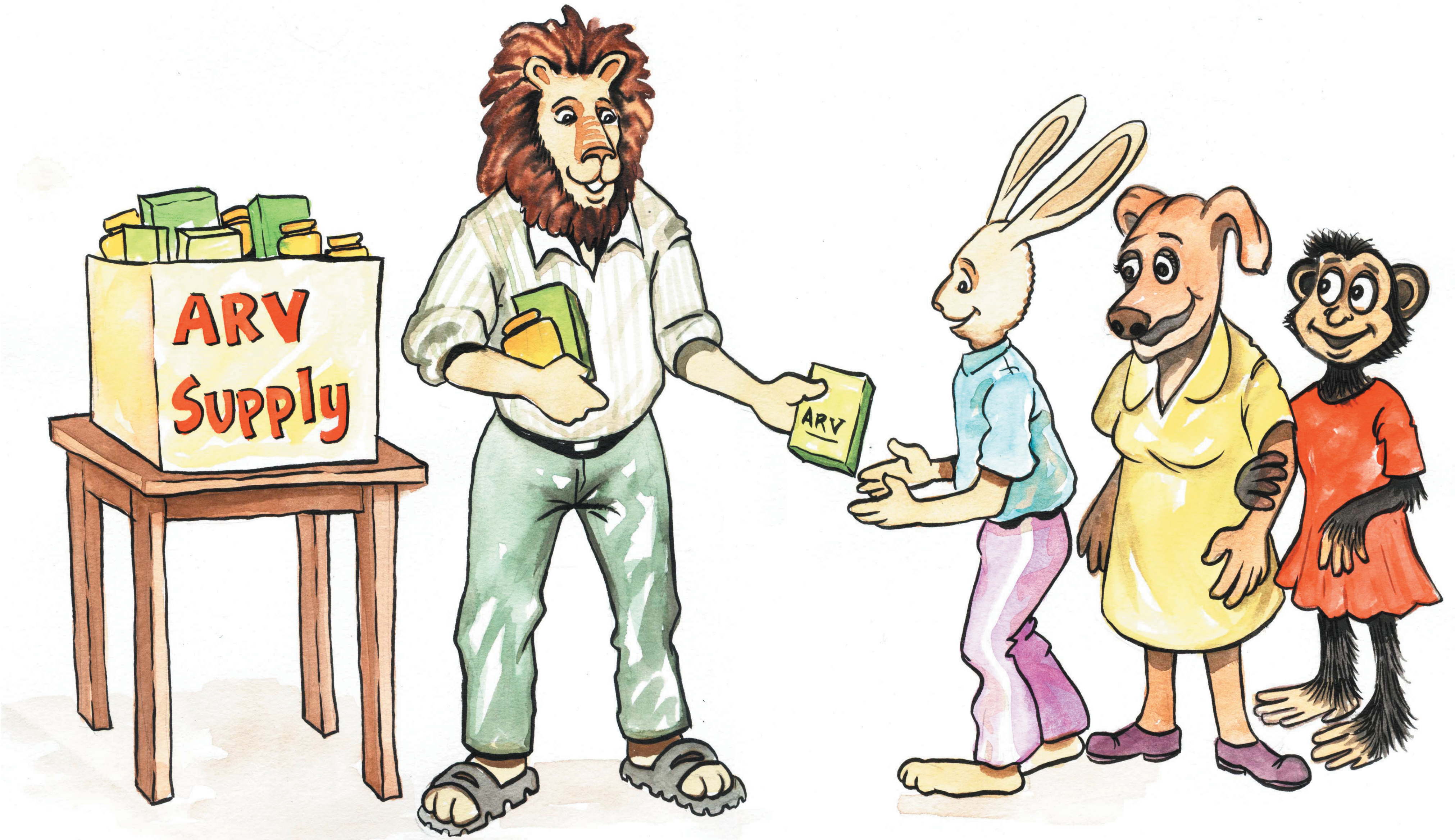

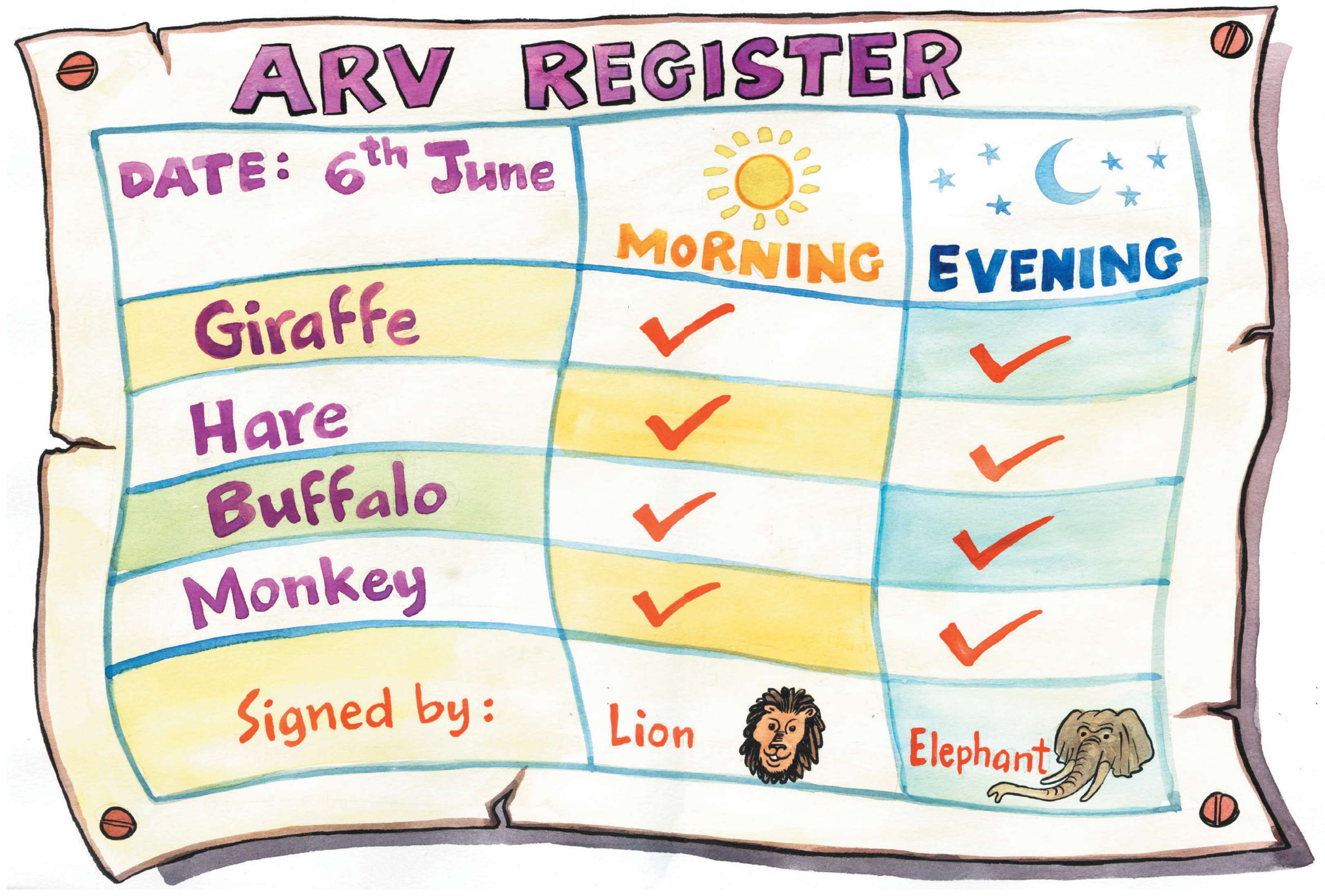

Uncle Lion and Auntie Elephant made sure that all the animals took their medicines twice a day, once in the morning and once in the evening. The ARV medicine stopped the Devimon Virus from destroying the Magic Force.

By eating properly and taking the ARV medicines the animals built up more Magic Force and the Devimon Virus n their bodies got fewer and fewer.

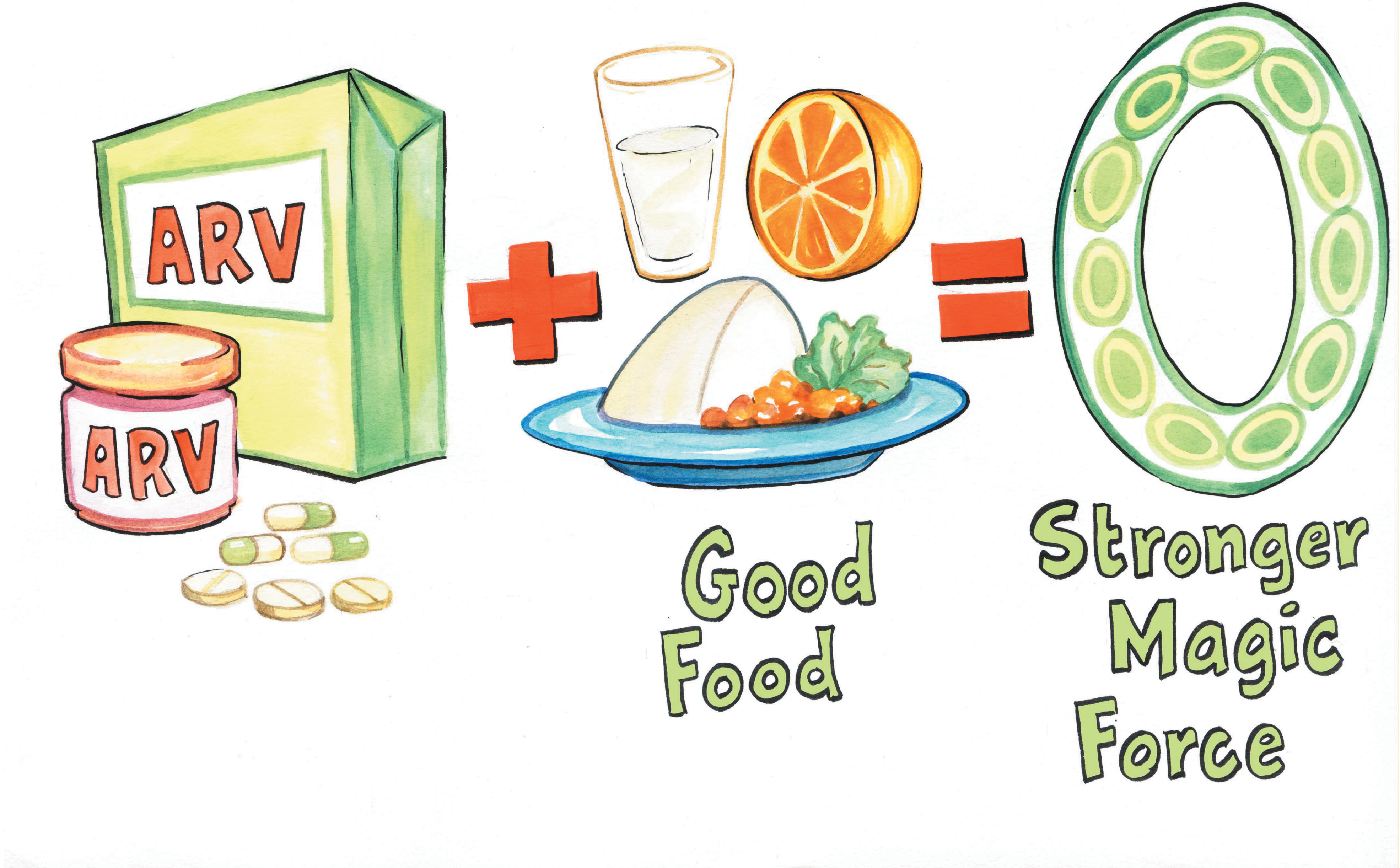

The animals were happy to take their medicines and began to get better. Some tablets were very big and difficult to swallow, so the animals dissolved them in water. Some medicines tasted horrible, so the animals took them with sweet drinks. All the animals looked after each other. Even the little animals were able to take care of a brother or sister or friend. This helped to build up their spirit and soon everyone in the village was happy.

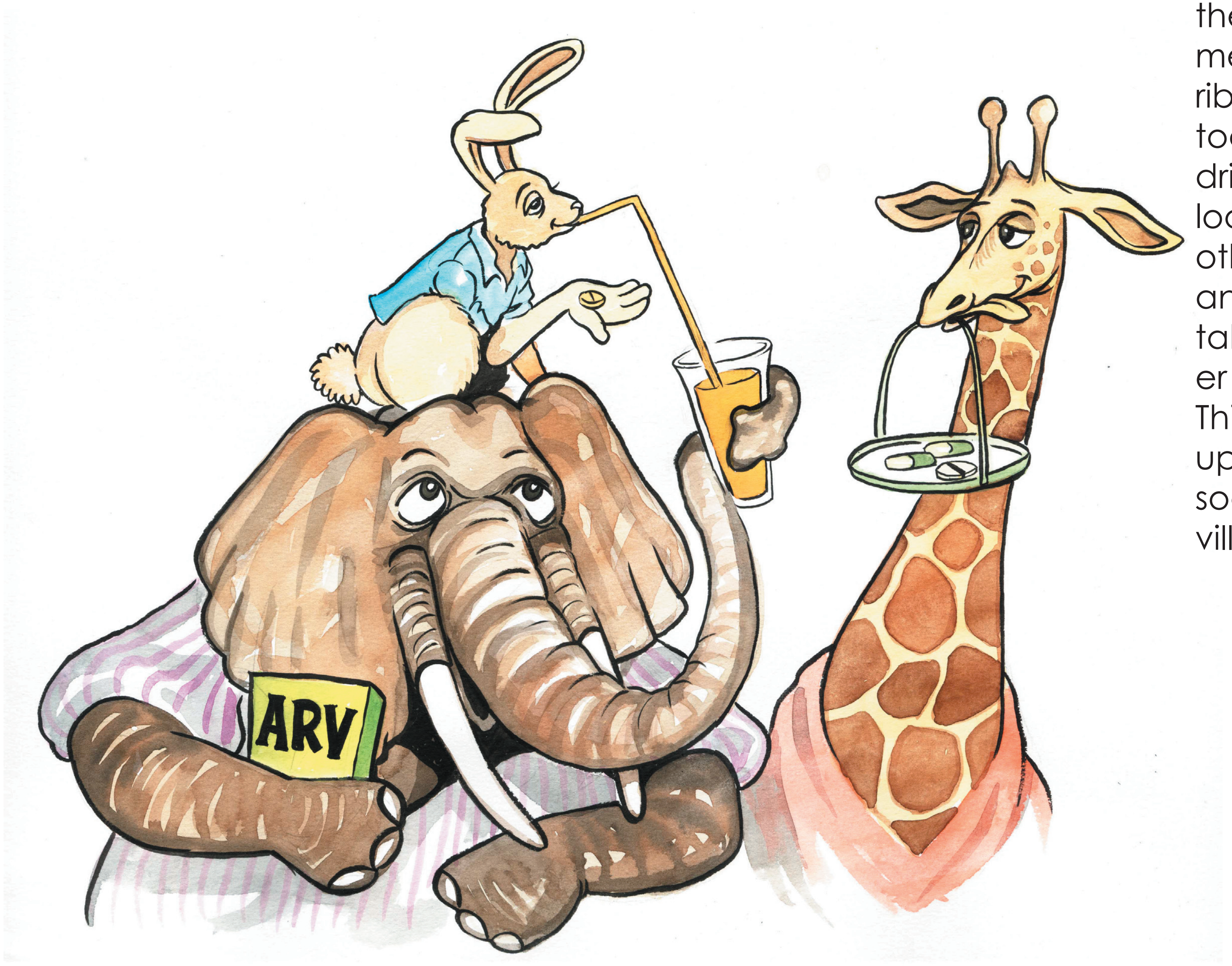

But the nasty Hyena never gave up. She cast a spell to make herself invisible so that the animals could not see her. The nasty Hyena whispered to the little animals to try to make them forget to take their medicines. She tried to blow the medicines away. She never stopped trying. She flew over the village on her broomstick going from house to house, but she never succeeded.

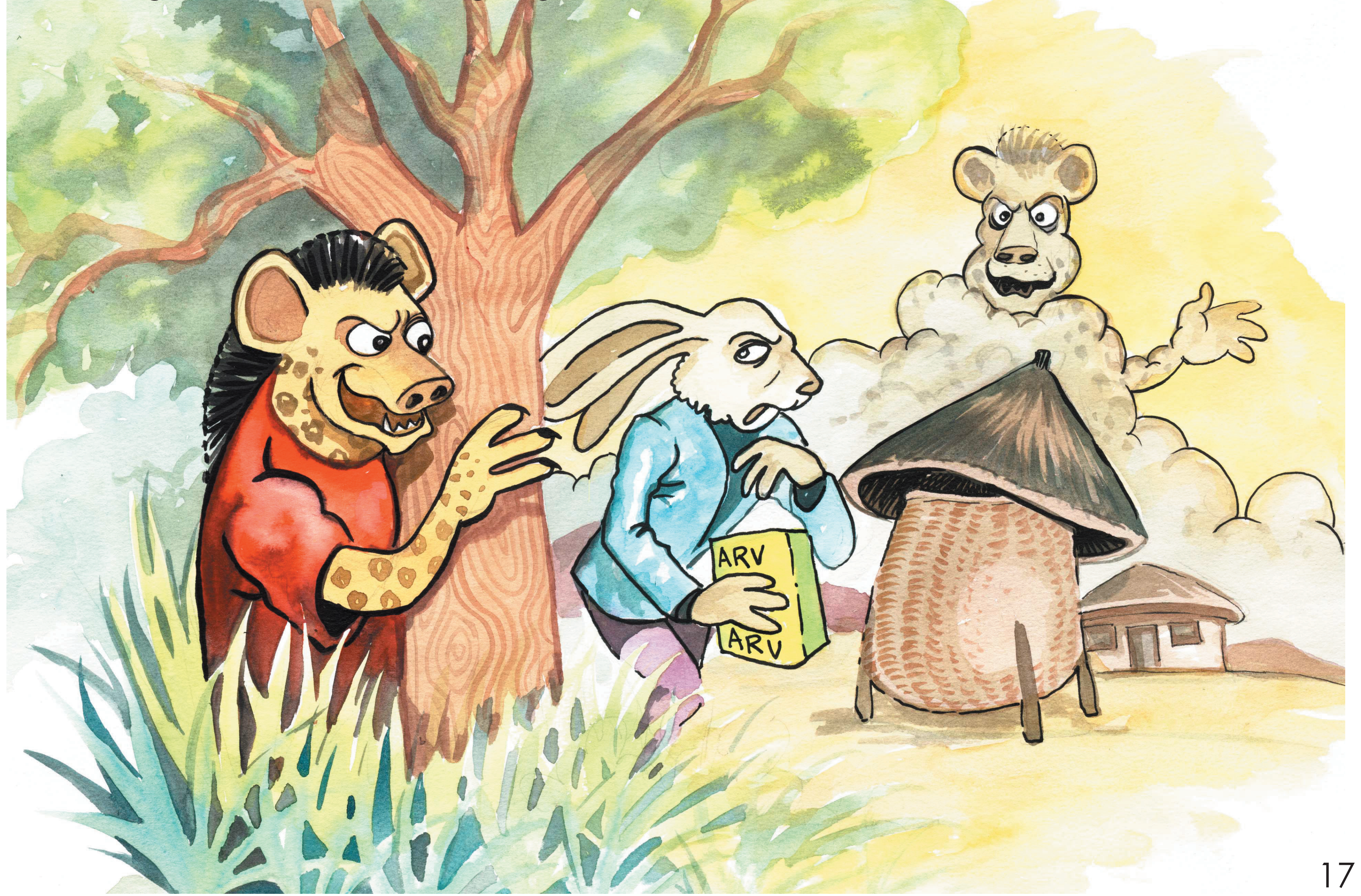

Do you know why? Because none of the animals forgot to take their medicines, and they all lived happily ever after.

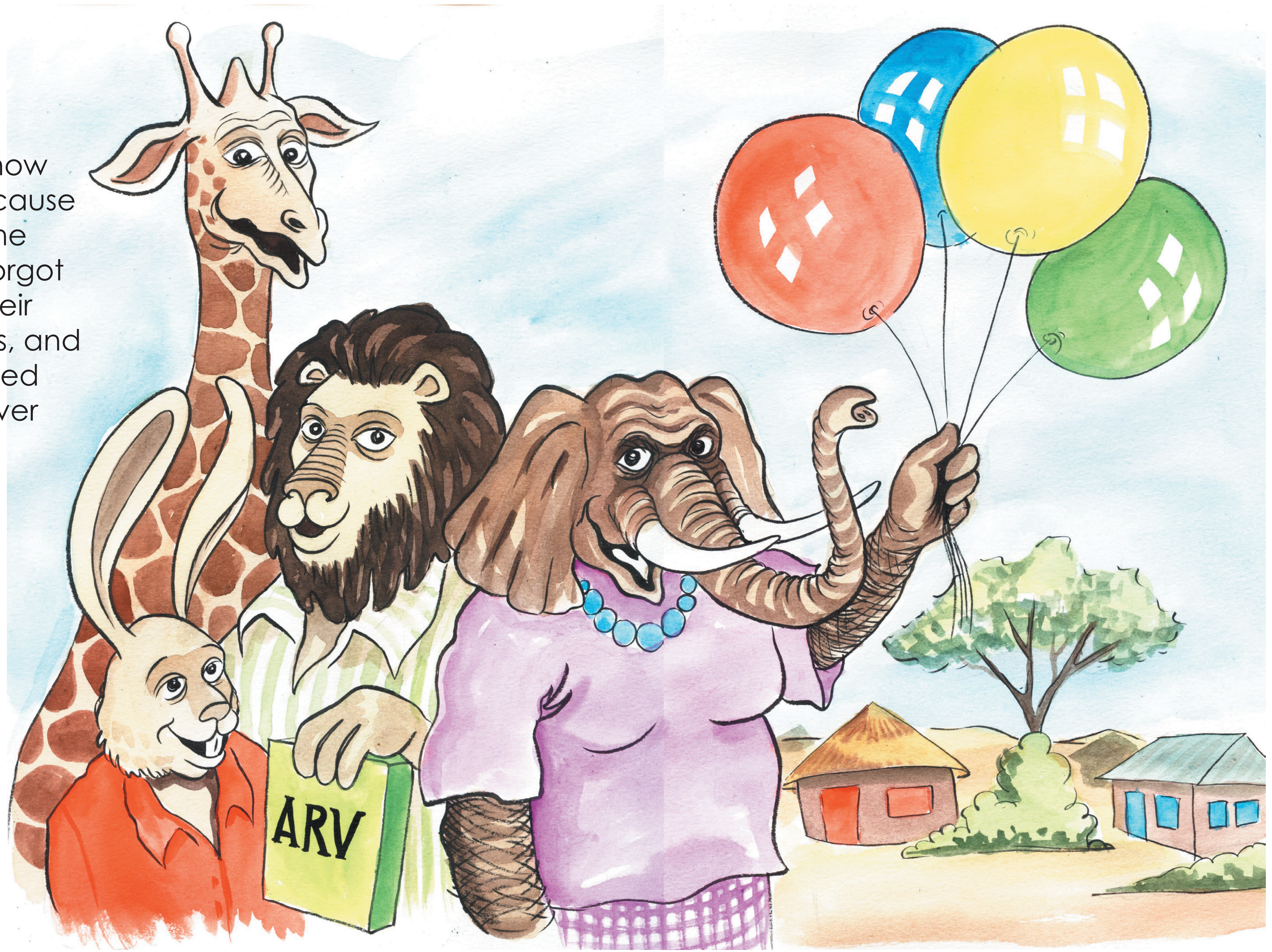

Questions about the story of the Devimon Virus.

1. How many characters are there in this story?

2. What characters do you like in this story?

3. What made the little puppy catch flu, the little monkey get diarrhoea, and Auntie Elephant get a fever?

4. What caused some animals to die?

5. What would have happened if Uncle Lion and Auntie Elephant had not discovered the pills called ARV?

6. What happened to the Devimon Virus when the little Lion took his pills correctly?

7. What are the ways to protect ourselves from the Devimon Virus suggested by Uncle Lion and Auntie Elephant?

Suggestions on how to use these questions to discuss HIV with children.

Children who are in primary school, aged 5-9 years old, may not know they have got HIV or may not be ready to accept that they are a person with HIV. However, the children should learn how to take good care of their health in order to fight diseases. Using questions 1-9 can help the children realize and accept the fact that although they have Devimon Virus in their bodies, they can be as healthy as other children if they know how to look after themselves.

You can use questions 10-19 to guide a discussion with children who are in secondary school, aged 10-13 years old, and who know they have HIV. Apart from using the story of the Devimon Virus, you could introduce the children to another HIV positive person or use newspaper articles that have both positive and negative messages to stimulate open discussion.
Examples of questions to be used in open discussions with children.

Q1. Is there anyone in your family who is sick at the moment or gets sick very often?

Q2. Who in your family looks after the sick person? Who in your family gives the sick person their pills?

Q3. How about you? Do you have an illness like little puppy, little monkey, or Auntie Elephant have the story?

Q4. What kind of medicine do you take?

Q5. If you were Uncle Lion, how would you explain about diseases?

Q6. If you were Uncle Lion, how would you care for the sick animals?

Q7. What would happen if you had the Devimon Virus in your body?

Q8. Do you think you have got the Devimon Virus in your body?

Q9. What would happen if you tried your best to fight the Devimon Virus? Do you know what you should do to fight the Devimon Virus?

Q10. Apart from Devimon Virus, do you know about any other viruses such as HIV Virus or AIDS Virus?

Q11.What do you know about the AIDS Virus?

Q12.Do you think the Devimon Virus is as dangerous as HIV Virus or AIDS Virus – or are all three of them the same?

Q13. What do you think the Devimon Virus is like? How does it grow or multiply?

Q14. What do you think the HIV or AIDS Virus is like? How does it grow or multiply?

Q15. What can you do to fight the Devimon Virus?

Q16. What can you do to fight the AIDS Virus?

Q17. Do you think it is true that people with AIDS can be healthy if they know how to take good care of themselves?

Q18. Do you think most people (such as friends, teachers at school, people who work in the market, people in your village or community) understand about AIDS? Do you think that you have a better understanding about AIDS than them?

Q19.There are still many people who have a bad attitude about AIDS. If you faced a problem because of someone's bad attitude about AIDS, how would you feel about that, and what would you do?

This fairy tale is written as a communication tool for use with children living with HIV and is aimed at creating a positive understanding of HIV.

Celestine Wamiru is an experienced illustrator and cartoonist based in Nairobi, Kenya. She can be contacted at the following address:
PO Box 9647-00100,
Nairobi, KENYA.
Email: cwamiru@yahoo.com
Telephone: +254 722 356824
